# Glymphatic system impairment in neurological disorders: potential mechanisms and therapeutic targets

**DOI:** 10.1186/s43556-026-00489-2

**Published:** 2026-06-12

**Authors:** Nan-Nan Wang, Zhi-Jun Liu, Yong Wang, Fei Cao, Da Xu

**Affiliations:** Department of Neurology, Union Hospital, Tongji Medical College, Huazhong University of Science and Technology, 1277 Jiefang Avenue, Wuhan, 430022 Hubei Province China

**Keywords:** Glymphatic system, Neurological disorders, Sleep–wake rhythm, Glial-vascular unit, Therapeutic approaches

## Abstract

The glymphatic system is a brain-wide metabolic clearance pathway, orchestrating the removal of neurotoxic wastes via glial-dependent perivascular networks. Mediated by polarized aquaporin-4 (AQP4) channels on astrocytic end-feet, this macroscopic system drives the convective exchange of cerebrospinal fluid (CSF) and interstitial fluid (ISF), establishing a functional coupling between the central nervous system (CNS) and the adaptive immune system. Emerging evidence highlights that glymphatic dysfunction act as both a consequence and a driver of numerous neurological disorders. Neurological pathologies, including neuroinflammation and gliovascular remodeling, compromise the structural and functional integrity of glymphatic architectures. Conversely, glymphatic dysfunction exacerbates neurotoxic wastes accumulation, accelerates disease progression, and perpetuates a pathological positive-feedback loop. Despite growing recognition of this bidirectional relationship, the precise mechanisms remain incompletely understood, and targeted therapeutic strategies are still lacking. In this review, we map the functional architecture of this pathway, from periarteriolar CSF influx to perivenous efflux, and dissect its dependence on critical modulators including sleep–wake rhythms, arterial pulsatility, and aging. Furthermore, we explore novel therapeutic interventions, ranging from AQP4-targeted pharmacological modulation to non-invasive physical approaches, and evaluate their potential to shift clinical paradigms from symptomatic management to disease modification.

## Introduction

**Fig. 1:**
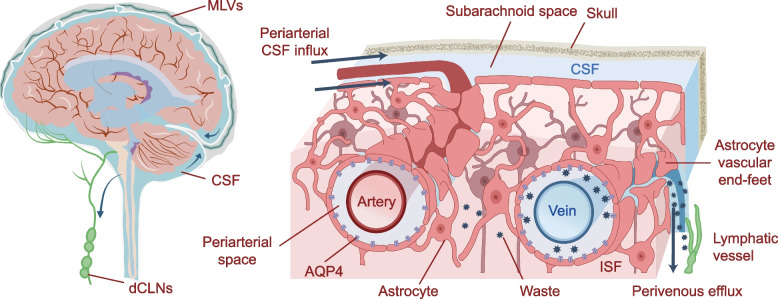
The process of glymphatic circulation, including production, exchange, and waste clearance

The maintenance of brain homeostasis is fundamentally dependent on fluid dynamics, which drives the continuous clearance of metabolic waste [1]. For decades, the prevailing dogma held that the brain lacked functional lymphatic vessels, attributing waste clearance solely to intracellular and extracellular mechanisms [2], including diffusion through the blood–brain barrier (BBB) and blood–cerebrospinal fluid barrier (BCSFB) [3], cellular degradation [4], and cerebrospinal fluid (CSF) absorption into the systemic circulation [5]. However, these mechanisms are insufficient to meet the brain’s high metabolic demands. This conceptual gap was bridged in 2012, when the Nedergaard team delineated a brain-wide pathway for central nervous system (CNS) fluid transport [6, 7]. Their finding revealed that CSF penetrates the brain parenchyma through perivascular spaces, where it undergoes convective exchange with interstitial fluid (ISF) before being eliminated along perivenous drainage routes. Utilizing in vivo two-photon imaging of small fluorescent tracers, researchers characterized this mechanism as a physiologically regulated system dependent on polarized aquaporin-4 (AQP4) channels on astrocytic end-feet [8] (Fig. 1). Given its functional analogy to the peripheral lymphatic system and its reliance on glial cells, this pathway was termed the glymphatic, or glial-lymphatic system [9].

Glymphatic perfusion of the brain exerts a multifaceted influence on neuro-pathophysiology. Primarily, it facilitates continuous metabolic waste clearance from the parenchyma into the CSF. Moreover, emerging evidence indicates that the glymphatic system serves as a conduit linking central and peripheral immunity [10]. Crucially, impairment of this system is a pathological hallmark of several neurological disorders, including Alzheimer’s disease (AD), Parkinson’s disease (PD), and epilepsy [11–14]. For instance, diffusion tensor image analysis along the perivascular space (DTI-ALPS) is a non-invasive MRI technique that indirectly assesses the glymphatic function by analyzing water diffusion within the brain's perivascular spaces [15, 16]. Studies have confirmed that glymphatic dysfunction, quantified by a diminished DTI-ALPS index, accelerates the progression of AD. This manifests as a heightened burden of neurotoxic aggregates, evidenced by elevated amyloid-beta (Aβ) and tau levels in CSF and on positron emission tomography (PET) imaging [17, 18]. Notably, pharmacological or genetic intervention of AQP4 polarization has been shown to alleviate AD-like pathology and rescue cognitive function, highlighting its potential as a therapeutic target [19, 20]. Similarly, impairing the glymphatic system exacerbates α-synuclein (α-syn) accumulation, drives dopaminergic neuronal loss, and hastens PD-like symptoms [21]. Furthermore, glial and vascular damages inherent to neurological insults frequently compromises glymphatic integrity, establishing a self-reinforcing positive feedback loop. In epilepsy, for instance, seizure-induced perilesional neuroinflammation disrupts the gliovascular unit [22], as reflected by a diminished DTI-ALPS index in the ipsilateral hemisphere relative to both the contralateral hemisphere and healthy controls [23]. Beyond acute inflammation, chronic seizure activity induces microvascular sclerosis within the epileptogenic focus [24, 25], which attenuates vessel pulsation-driven CSF flow and exacerbates glymphatic stasis [26]. Mitigating secondary complications of epilepsy, such as cerebral edema, may restore glymphatic function [27].

While glymphatic system revolutionized our conceptualization of cerebral clearance and unveiled promising clinical frontiers, critical gaps persist. Specifically, the underlying mechanisms remain obscure, standardized diagnostic methods are limited, and the clinical translation of these insights remains elusive. In this review, we delineate the fundamental architecture of the glymphatic system, and provide a detailed elucidation of the physiological and pathological mechanisms of its dysfunction. Furthermore, we synthesize glymphatic dysfunction across neurological disorders, covering pathogenic mechanisms, experimental and imaging methodologies, and emerging therapeutic strategies, with a particular emphasis on clinical translation.

## The structure and function of the glymphatic system

The glymphatic circulation is a highly organized fluid transport network, which has been extensively characterized in animal models [28, 29]. This section provides a comprehensive overview of these anatomical features and the regulatory landscape governing glymphatic kinetics.

### Key anatomical components: perivascular spaces and astrocytic end-feet

The glymphatic system is a brain-wide perivascular network constituted by the provascular spaces surrounding blood vessels and the end-feet of astrocytic glial cells [30]. The BBB, characterized by specialized tight junctions between endothelial cells [31], is functionally and structurally integrated with the glymphatic system through the shared astrocytic end-feet and their AQP4 channels. By precisely regulating solute exchange between the systemic circulation and the ISF, the BBB serves as a selective interface that restricts the entry of toxins, pathogens, and other harmful substances, maintaining the homeostatic milieu requisite for efficient glymphatic circulation [32].

Mechanistically, CSF from the subarachnoid space gains entry to the brain through the perivascular spaces (PVS) of large arteries [33], moves distally as these vessels branch into penetrating arterioles, and ultimately reaches the parenchyma [34], where it mixes with ISF. At the parenchymal interface, AQP4 channels, located at astrocytic end-feet act as low-resistance conduits for fluid exchange between perivascular and interstitial compartments [35, 36], facilitating the coupled processes of para-vascular inflow and convective solute clearance. Ultimately, the combined CSF-ISF fluid is channeled through several distinct anatomical routes, including the perineural sheaths of cranial and spinal nerves, meningeal lymphatic vessels, and arachnoid granulations [37]. These pathways converge into the cervical lymphatic network, eventually draining into the systemic circulation.

### CSF–ISF exchange dynamics and AQP4 channels

Fluid exchange between CSF and ISF involves two primary mechanisms: diffusion and convection. Historical models emphasized diffusion as the dominant mechanism [38] for intraparenchymal transport. However, current evidence positions CSF-ISF exchange as predominantly convective, which is driven by arterial pulsations and mediated by AQP4 channels within a multi-compartmental coupled system [10, 39]. Arterial pulsations generate periodic pressure gradients that propagate along cerebral arteries, driving net forward CSF flux within periarteriolar spaces, a mechanism known as perivascular pumping [40–42].

Additionally, polarized AQP4 proteins, occupying 20–60% of the perivascular membrane adjacent to the vessel wall [43], acts as the primary exchange interface. AQP4 is a homotetramer composed of AQP4 monomers, featuring two predominant isoforms: AQP4-M1 and AQP4-M23 [44]. Each monomer spans the membrane six times, with three extracellular and two intracellular loops folding into an hourglass pore [45, 46]. On the astrocytic membrane, AQP4 tetramers form orthogonal arrays of particles (OAPs) [47], whose size is regulated by the ratio of M1 to M23 isoforms. Simultaneously, AQP4 polarization and channel function require assembly with other membrane proteins such as the dystrophin-glycoprotein complex (DGC) [48]. AQP4 dysfunction, whether through inhibition [49], mis-localization [50], or depolarization [51], correlates with toxic protein accumulation and accelerated pathology in neurodegenerative disease models. AQP4-targeted interventions represent a promising therapeutic strategy for glymphatic modulation, as shown in Table 1. Their clinical applications will be presented in the "Potential Therapeutic Strategies" section.

**Table 1:** AQP4-targeted interventions

Types	Levels of Interventions	Interventions	Application contexts	Reference
Pharmacological Interventions	Transcriptional Level	Trolox	AQP4 upregulation enhances the integrity of the BBB and reduces perihematomal edema following intracerebral hemorrhage	[52]
		BAY11-7082	Reducing the expressions of AQP4 in OGD/R astrocytes lead to BBB injury following cerebral I/R	[53]
	Post-transcriptional	miR-130a Antagomir	Preventing cerebral edema mediated by ischemia–reperfusion injury	[54]
		miR-145	Preventing cerebral ischemia–reperfusion injury by targeting AQP4	[55]
	Phosphorylation	CaMKII	Reducing brain edema and improves BBB after cerebral ischemia injury	[56]
	Membrane Localization	Trifluoperazine	Diminishing astrocyte activation in, ablating astrocyte activation, and accelerating functional recovery	[57]
	AQP4 Polarization	Melatonin	Mitigating sleep restriction-induced cognitive and glymphatic dysfunction	[58]
	Channel Blockade	TGN-020	Exacerbating tau propagation in AD models by inhibiting AQP4 protein function	[59]
Gene Interventions	Gene Expression	Lentivirus Packaging	The increased expression of AQP4 reduces astrocyte viability and leads to cellular edema	[60]
		siRNA	Inhibiting long-term learning and working memory abilities	[61]
Physical Interventions	AQP4 Polarization	High-intensity Interval Training	Ameliorates AD-like pathology by modulating the polarization of AQP4	[19]
	Membrane Localization	Low-Intensity Ultrasound	Promoting glymphatic influx and clearance	[62]

AQP4, Aquaporin 4; BBB, Blood–brain barrier; OGD/R, Oxygen–glucose deprivation/Re‑oxygenation; I/R, Ischemia/Reperfusion; CaMKII, Calcium/Calmodulin-dependent protein kinase II; siRNA, Small interfering RNA; AD, Alzheimer's disease

### Regulation of glymphatic system by physiological factors

**Fig. 2:**
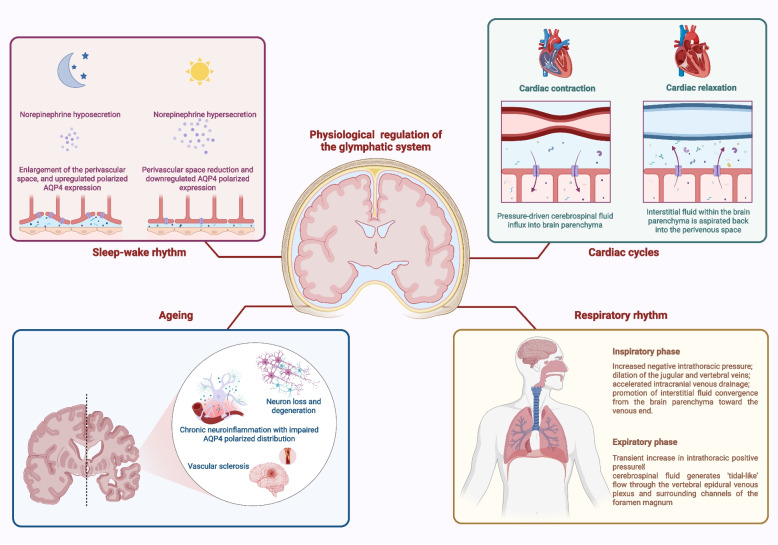
Physiological regulatory factors of the glymphatic system include sleep–wake rhythm, cardiac cycle, aging, and respiratory rhythm

The physiological regulation of the glymphatic system is a multifactorial and dynamically integrated process [63] (Fig. 2). While the sleep–wake cycle serves as its primary rhythmic modulator [64], physiological brain pulsations provide the requisite mechanical driving forces [65]. Notably, aging and glymphatic dysfunction form a self-perpetuating vicious cycle that functions as a critical pathological driver of neurodegeneration [66]. Critically, these regulatory elements are not isolated but exhibit intricate crosstalk. For example, aging-associated sleep fragmentation impairs glymphatic efficiency, while the sleep state itself robustly augments the physiological pulsations that facilitate fluid flux.

#### Sleep–wake rhythm

Previous studies have demonstrated that, compared to wakefulness, glymphatic activity is enhanced during anesthesia and sleep, especially during non-rapid eye movement (NREM) sleep [67, 68]. Conversely, sleep deprivation suppresses this convective exchange, resulting in the sequestration of metabolic waste and the disruption of cerebral proteostasis [69, 70]. Although not fully elucidated, the underlying mechanisms are thought to be multifactorial. NREM sleep is characterized by periodic silent periods of norepinephrine (NE) release from the locus coeruleus (LC) [71, 72]. This neuromodulatory quiescence acts as a primary driver of slow-wave vasomotion, which subsequently orchestrates the pulsatile fluid flux essential for glymphatic clearance during natural sleep [73, 74]; Recent evidence also suggests that glymphatic clearance efficiency peaks during the slow-wave sleep (SWS) phase of NREM [75]. This phase is marked by increased δ-wave (0.5–4 Hz) power, which correlates positively with CSF inflow and clearance [76, 77]; Importantly, the polarized expression of AQP4 is also influenced by sleep–wake rhythm [78], through which sleep fragmentation impairs cognitive behaviors, particularly learning and memory [79]. Therefore, modulating sleep-specific neuro-oscillations, rather than simply extending total sleep time, emerges as a potent therapeutic strategy. Melatonin, an endogenous circadian regulator [80], has recently been demonstrated to alleviate cognitive deficits and behavioral abnormalities following intracerebral hemorrhage by reshaping sleep architecture and glymphatic circulation [81].

Sleep disturbances frequently precede the onset of various neurological diseases and serve as an early indicator of dementia. Current hypotheses posit that the sleep–wake rhythm may function as a fundamental nexus between glymphatic dysfunction and neurological disorders. Consistent with this, research revealed that melatonin could improve depression-like behaviors by upregulating the circadian clock protein Per2, which in turn restore the rhythmic polarization of AQP4 [82].

#### Physiological brain pulsations

The consistent, directional flow of glymphatic circulation is mainly driven by the synchronized pulsations of cerebral arteries, which are governed by cardiac cycles, vasomotor activity, and respiratory rhythms. The direction of glymphatic flow aligns with the direction of cerebral blood flow, and exhibits a frequency similar to the cardiac cycle [83]. Physiological cardiac systolic rhythm and contractile function create a blood pressure pulse that propagates through cerebral vessels [84]. This localized pressure wave travels along the arterial walls, creating the mechanical displacement necessary to drive CSF from the periarterial spaces into the dense neural parenchyma [85]. Conversely, cardiac arrhythmias compromise vascular wall wave dynamics and disrupt the periodicity of arteriolar pulsations [86], reducing both the velocity and rhythmicity of glymphatic circulation. In the context of heart failure, the impairment of cerebrovascular reactivity and neurovascular coupling further destabilizes CSF flow kinetics [87]. Consequently, the restoration of cardiac rhythm and the maintenance of optimal cardiac output may be useful. For example, in patients with atrial fibrillation (AF), catheter ablation to restore sinus rhythm could boost the DTI-ALPS index, potentially preventing AF-related cognitive decline and dementia [88]. Beyond cardiac-driven forces, cerebral vessels exhibit intrinsic, low-frequency rhythmic oscillation that operate independently of the cardiac cycle. These pulsations arise from the spontaneous contractile-relaxation activity of pericytes and perivascular fibroblasts [89, 90]. This low-frequency activity functions as a localized micro-pump, directly propelling CSF flux and facilitating efficient exchange with the ISF [91]. However, cerebral vasculopathies, such as atherosclerosis and vascular malformations, diminish arterial elasticity and reduce vascular wall compliance. These structural alterations compromise vasomotor function and attenuate the pulsatile power necessary for glymphatic transport, potentially providing a mechanistic link to the elevated risk of neurodegenerative diseases.

Moreover, high-resolution real-time MRI studies in humans have observed that the deep inspiratory breath, referred as diaphragmatic breathing or deep breathing, could augment the cranial displacement of CSF and accelerate the rate of CSF–ISF exchange [92]. Mechanistically, this process is mediated by the periodic compression and decompression of the perivenous spaces, driven by respiratory-induced pressure gradients [93]. Inhalation creates intrathoracic negative pressure, collapsing the intracranial venous plexus to expand perivenous spaces and generate suction. Exhalation increases abdominal pressure, causing vertebral venous reflux to compress these spaces and produce propulsion. Collectively, this establishes a dynamic pressure gradient for unidirectional outflow of waste-laden ISF toward cervical lymphatics. Although the respiratory contribution is relatively modest, these findings delineate a novel therapeutic paradigm, suggesting that interventions such as continuous positive airway pressure (CPAP) could be leveraged to enhance glymphatic clearance [94].

#### Aging

Age-related functional and structural deterioration compromises the entire glymphatic system, transforming it from a robust fluid-wash cycle to an inefficient clearance mechanism with drastically reduced waste-clearance efficiency. At the structural level, age-related cardiac dysfunction and vascular stiffening impair the pumping action [95]; The nasopharyngeal lymphatic plexus is a major outflow route for CSF into the deep cervical lymph nodes [96]. This plexus undergoes progressive atrophy with aging, coinciding with enhanced type I interferon signaling and elevated levels of other pro-inflammatory cytokines [97]. Aging also promotes morphological atrophy and functional impairment in astrocytes, disrupting the polarized perivascular localization of AQP4 [98].

Furthermore, age-related attrition across multiple physiological regulatory systems exacerbates glymphatic dysfunction. For instance, senescence is characterized by a profound degradation of sleep architecture, including prolonged sleep latency, increased fragmentation with frequent arousals, and a precipitous decline in SWS in favor of lighter NREM stages [99, 100]. On the other hand, impaired glymphatic circulation triggers a proteotoxic cascade of metabolite accumulation, which accelerates secondary brain injury and drives premature cerebral senescence. Consequently, a multi-target therapeutic paradigm, integrating the modulation of sleep oscillations, vascular preservation, and the restoration of fluid dynamics, will be a potent strategy for neuroprotection and the mitigation of neurodegenerative progression.

## Mechanisms underlying glymphatic dysfunction

**Fig. 3:**
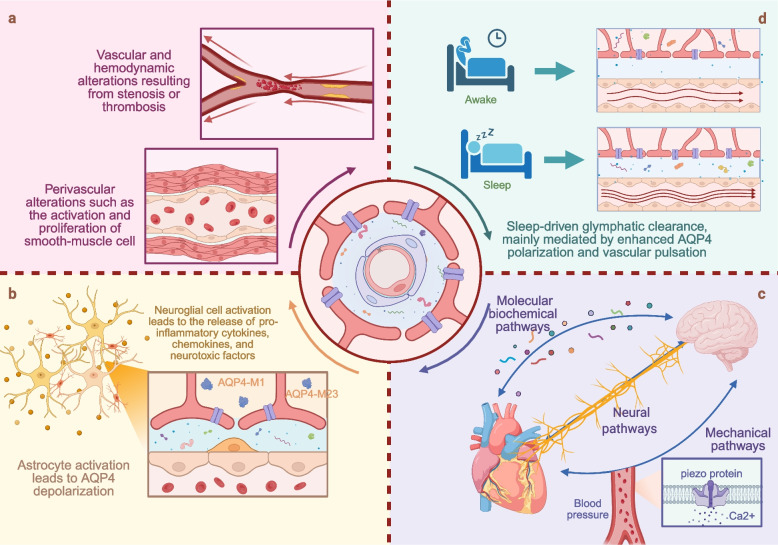
Principal mechanisms underlying glymphatic dysfunction. **a** Neuro-gliovascular unit dysfunction. **b** Neuroinflammation and glial activation. **c** Heart-brain axis dysfunction. **d** Sleep disturbances and circadian dysregulation

As previously discussed, the glymphatic system is pivotal for cerebral homeostasis and is implicated in the pathogenesis and progression of neurological disorders. Subsequently, we will illustrate the mechanisms underlying its dysfunction (Fig. 3).

### Neuro-gliovascular unit dysfunction

Evidence has established that neuronal activity and cerebral blood flow are tightly coupled and precisely regulated [101]. This relationship, termed neurovascular coupling, facilitates a localized increase in regional blood flow in response to elevated neural activity. This physiological mechanism ensures that metabolic supply is strictly aligned with functional demand, underscoring the principle that cerebral homeostasis depends on the integrated communication within the neuro-gliovascular unit (NGVU). NGVU is a multicellular structure composed of neurons, glial cells, perivascular cells, and the perivascular space [102]. It is integral to regulating cerebral blood flow, angiogenesis promotion, neurotoxic waste clearance, and preservation of BBB integrity. Under physiological conditions, endothelial cells transduce mechanical shear stress into biochemical signals, releasing paracrine factors that modulate astrocytic activity. This activation prompts astrocytes to secrete vasoactive factors that stimulate pericyte proliferation, modulate vascular constriction and dilation, and ultimately govern blood flow as well as glymphatic circulation. Concomitantly, physiological activation of astrocytes increases AQP4 polarization, and pericytes tether AQP4 to the end-foot membranes, promoting the influx of glymphatic fluid into the brain parenchyma [103]. Consequently, the structural and functional decoupling of NGVU precipitates the collapse of glymphatic circulation, which frequently precedes detectable brain pathology. In the following section, we provide a detailed analysis of each NGVU component and its underlying regulatory mechanisms, as summarized in Table 2.

**Table 2:** The roles of each component of the neuro-gliovascular unit in glymphatic circulation

Components		Location/ Characteristics	Functions
Perivascular cells	Endothelial cells	The innermost layer of blood vessels	Constituting the circulation pathways of blood and glymphatic fluid
	Pericytes	Between the basement membrane and endothelial cells of capillaries	Possessing contractile capacity, regulating blood flow and the speed of glymphatic circulation
	Perivascular Macrophages	Perivascular Space	Immune defense and clearance, clearing metabolic waste within glymphatic circulation and promoting its excretion
Glial cells	Astrocyte	The end-feet cover the surface of blood vessels	Constituting boundary of Perivascular Space; The AQP4 protein is the channel for CSF-ISF exchange
	Microglia	Brain parenchyma	Immune defense and clearance
Extracellular matrix		Pericellular	The viscosity is related to the resistance of lymphatic circulation
Neurons		Brain parenchyma	Generating neural signals and ion waves to drive the flow of blood and lymph

AQP4, Aquaporin 4; CSF, Cerebrospinal fluid; ISF, Interstitial fluid

#### Vascular and hemodynamics alterations

As established, the vascular system provides a comprehensive brain-wide scaffold for glymphatic transport, driven by the rhythmic oscillations of vascular tone. Recent evidence further demonstrates that slow-wave neural activity and fluctuations in cerebral blood volume are precisely synchronized with CSF dynamics within the fourth ventricle [104]. Fluctuations in cerebral blood volume and arterial diameter have been shown to synchronize with rhythmic fluctuations in both neural activation and norepinephrine levels [105]. In mice, experimental whisker stimulation that drives functional hyperemia could also potentiates glymphatic influx and clearance [106].

Maintaining vascular health and adequate cerebral blood flow is crucial for sustaining efficient glymphatic system function. However, various pathological conditions, including aging, hypertension, and atherosclerosis, impair the integrity of this regulatory mechanism. For example, chronic elevation of blood pressure drives hyalinization and fibrotic thickening in cerebral small vessels, ultimately impairing the barrier integrity and contractility of vascular [107]. These disruptions frequently arise as primary initiating events, manifesting long before the appearance of overt histopathological lesions. For instance, following cerebral ischemia, the activation of the p38 MAPK/MMP-9 signaling cascade in microglia compromises endothelial cell integrity, disrupting glymphatic efflux pathways [108]. Nevertheless, such alterations are not readily detectable using conventional imaging examinations. Future research should prioritize functional and metabolic brain examinations, as well as biomarker detection, to enable early detection and timely intervention.

#### Perivascular cells alterations

In addition to direct vascular regulation, perivascular cells mediate precise local control of glymphatic circulation. Smooth muscle cells (SMCs) and pericytes, collectively termed vascular mural cells [109], line the entire cerebrovascular system and segregate into discrete microvascular compartments. The contraction and relaxation of concentric SMC rings produce the primary mechanical force that drives glymphatic flow. Following endothelial injury, SMCs undergo a phenotypic transition to a migratory-proliferative state in response to diverse noxious stimuli, encroaching on the intimal layer to form the neointima and thereby impairing hemodynamic regulation [110]. Pericytes are the predominant mural cells of the capillary bed, extending extensive circumferential processes along the lumen and expressing α-smooth muscle actin (α-SMA) [111] which modulates blood flow through contractile activity. Beyond their hemodynamic roles, pericytes preserve AQP4 polarization by employing agrin to stabilize its interaction with dystrophin, tethering of this protein complex to laminin within the basement membrane (BM) [112]. The conditional knockout of the pericyte-specific cyclin-dependent kinase 5 (CDK5) gene disrupts AQP4 polarization [113]. Importantly, the loss or dysfunction of pericytes has been documented across a spectrum of neurological disorders, such as AD, epilepsy, and stroke [114–116]. Furthermore, pericyte injury drives glymphatic dysfunction, exacerbating the progression of neurological disorders. Collectively, these interactions form a reciprocal feedback loop that unites structural integrity, functional homeostasis, and pathological progression.

Perivascular macrophages (PVMs), a distinct embryonic-derived immune cell population, resides within the PVS. Strategically positioned at the brain–vascular interface, PVMs lie tightly apposed to intracranial vessels between the vascular basement membrane and the pia mater, which are in direct contact with CSF and function to phagocytose metabolic waste [117]. Additionally, PVMs secrete mesencephalic astrocyte-derived neurotrophic factor (MANF) [118], which acts distally to suppress astrocytic stress responses and sustain the polarized localization of AQP4. However, upon detecting neuroinflammatory signals, PVMs become activated and releasing pro-inflammatory cytokines. Moreover, aging impairs the phagocytic and degradative functions of PVMs [119], leading to an accumulation of undegradable material and their conversion into waste-laden senescent cells, which are ultimately replaced by monocyte-derived macrophages. These monocyte-derived macrophages display an elevated pro-inflammatory propensity [120]. Therefore, the functional decline of PVMs is not merely a symptom of cellular aging but a critical driver of the subsequent impairment to the brain's waste-clearance system.

#### Impaired AQP4 polarization and astrocytic dysfunction

AQP4 exists as two major isoforms, M1 and M23, generated via alternative translation initiation from distinct start codons. The AQP4-M1 isoform is highly mobile in the plasma membrane and facilitates CSF circulation, whereas the AQP4-M23 isoform assembles into OAPs, a structural arrangement essential for establishing AQP4 polarization [121]. Key proteins such as syntrophin alpha 1 (SNTA1) [122] and the dystroglycan complex (DG) [123] tether AQP4 to astrocytic end-feet via direct protein–protein interactions. Perturbations in AQP4 expression, post-translational modification, or subcellular localization disrupt this polarization, with pathological consequences detailed in Table 1.

Astrocytes are generally classified into two functional subtypes: neurotoxic A1 and neuroprotective A2 phenotypes [124]. Notably, A1 astrocytes exhibit loss of AQP4 polarization, whereas A2 astrocytes preserve polarized AQP4 distribution [125]. The exact mechanism remains unclear but may involve the disruption of the AQP4-anchoring complex [126, 127]. Accumulating evidence indicates that A1 astrocyte activation predominates in most neurological disorders, and skewing the astrocytic phenotype from A1 toward A2 may confer therapeutic benefits [128]. We hypothesize that this phenotypic switch may be related to the depolarization and repolarization of AQP4.

### Heart-brain axis dysfunction

The nervous and cardiovascular systems are anatomically and functionally integrated, jointly regulating essential physiological processes, including human physiology, cognition, and emotional states. Various neurological disorders, including stroke [129], epilepsy [130], and brain tumors [131], frequently associate with cardiac injury and electrocardiogram abnormalities, such as prolonged QTc intervals and left ventricular diastolic dysfunction. Reciprocally, cardiac function directly influences essential cerebral physiological activities. Existing studies have confirmed that glymphatic clearance is impaired in patients with heart failure [132]. These reciprocal connections constitute the "brain–heart axis." We hypothesize that pathological insults disrupt this neurocardiac circuit, consequently impairing glymphatic function. However, the specific mechanisms remain poorly elucidated. To address this gap, we illustrated three primary pathways: neural, mechanical, and molecular biochemical.

#### Neural pathways

The central autonomic network (CAN) mediates bidirectional communication between supraspinal centers and the heart, enabling precise neurocardiac regulation [133]. Additionally, the heart houses an intrinsic cardiac nervous system that relays afferent feedback to the brain through spinal nerves and vagal afferents, which project to CAN-containing brain regions.

CNS and cardiovascular diseases trigger acute and chronic stress responses, provoking excessive catecholamine release. Via this pathway, sustained catecholamine exposure hyperactivates adrenergic receptors, driving persistent elevations in ventricular contractility, heart rate, and cardiac output. At the same time, systemic vasoconstriction and increased venous tone may elevate the risk of cardiac complications, including myocardial cell necrosis and coronary artery spasms. Within the brain, sympathetic overactivation induces cerebral cerebral vasoconstriction and disrupts neurovascular coupling, fostering neuroinflammation via NLRP3 inflammasome activation and reactive gliosis [134]. Collectively, these alterations contribute to neurological deficits and impaired driving force behind the glymphatic circulation.

Future treatments should target the entire bidirectional cardiocerebral axis, restore the balance between the sympathetic and parasympathetic nervous systems, and disrupt heart-brain vicious cycle. Potential therapeutic interventions include neuromodulation approaches, including vagus nerve stimulation and carotid sinus stimulation, as well as pharmacological approaches designed to augment parasympathetic tone and mitigate aberrant sympathetic hyperactivity.

#### Mechanical pathways

Cardiac pulsations propagate through the cerebral vascular system, generating rhythmic intracranial pressure pulsations. In response to fluctuations in arterial blood pressure, stretch-sensitive baroreceptor within the aortic arch and carotid sinus walls transduces arterial pressure variations into neural signals, mediating the baroreflex [135]. This reflex regulates heart rate, myocardial contractility, and peripheral vascular resistance. Scientists refer to the heart rate oscillations (HRO) as a manifestation of this mechanism, which is evoked by arterial pressure pulsations and transduced by central baroreceptors [136]. Subsequently, we will focus on the underlying mechanotransduction pathway, particularly the role of Piezo1 channels in mediating this heart-brain communication.

Piezo1 is a mechanically activated cation channel functionally expressed in the endothelial cells. Upon sensing hemodynamic mechanical forces, its mechanical activation triggers extracellular Ca^2+^, Na^+^, and K^+^ influx, influencing endothelial cell morphology and vascular architecture [137], and ultimately orchestrating vascular development, function, and maintenance. Intriguingly, recent studies have discovered that Piezo1-mediated mechanical signals can propagate to astrocytes in a layer-by-layer manner, generating propagating calcium waves. Upon Piezo1 inhibition or knockdown, the responsiveness of astrocytes to CSF flow is rapidly attenuated. Animal experiments further demonstrated that Yoda1, a selective Piezo1 agonist, can rescues multiple components of the glymphatic-lymphatic system in diseased and aged mice, including meningeal lymphatic circulation, drainage to the deep cervical lymph nodes, and CSF perfusion within the brain [138].

Further strategies are warranted to advance our understanding of mechano-transduction pathways and promote mechanoregulation-based integrated management of the cardiocerebrovascular system.

#### Molecular biochemical pathways

Bidirectional brain–heart crosstalk is further coordinated by a signaling network that integrates systemic humoral circulation with local paracrine signaling governed by a spectrum of bioactive molecules, including hormones and inflammatory mediators.

The hypothalamic–pituitary–adrenal (HPA) axis, tightly linked to hormonal dysregulation, is primarily regulated by the hypothalamic paraventricular nucleus. This nucleus releases key regulators such as corticotropin-releasing hormone and vasopressin. Under stressful conditions, these hormones act on the pituitary gland to improve adrenocorticotropic hormone (ACTH) release [139]. As previously discussed, overactivation of the HPA axis drives excessive sympathetic outflow, resulting in neural pathway dysfunction. Additionally, ACTH stimulates the adrenal cortex to secrete the steroid hormone cortisol. This elevated cortisol, when sustained at high levels, exerts detrimental neurotoxic and cardiotoxic effects. Research has reported that elevated cortisol levels were linked to increased cerebral amyloid deposition [140] and higher post-stroke mortality [141]. Furthermore, even in the general population, cortisol levels measured in blood, urine, or hair show positive correlations with a higher burden of cardiovascular risk factors and increased mortality rates [142].

Local injury elicits acute and chronic inflammatory responses that drive the release of pro‑ and anti‑inflammatory mediators into the systemic circulation. These inflammatory factors activate downstream signaling pathways, culminating in remote organ damage. A compelling clinical example of this systemic crosstalk is observed in stroke, a condition commonly referred to as cerebral-cardiac syndrome (CCS). Ischemic stroke is the leading cause of CCS, with over 90% of patients developing cardiac complications including arrhythmia and cardiac arrest [143]. Accumulating evidence suggests that this phenomenon may be related to the systemic inflammatory response post-stroke. The peripheral immune response to stroke is initiated within minutes of onset, driven by damage-associated molecular patterns (DAMPs) released from injured tissue in the ischemic brain and recognized by dying cells and other immune cells [144]. In murine shock models, early local neuroinflammation following ischemia drives subsequent cardiac impairment, primarily through glial cell activation and the induction of inflammatory signaling cascades, including NF-κB, NLRP3, and JAK-STAT [145]. Local and systemic immune responses represent a strategic frontier for developing therapeutic interventions.

### Neuroinflammation and glial activation

Neuroinflammation is characterized by the activation of glial cells, particularly microglia and astrocytes, which triggers a signaling cascade mediated by the release of chemical signals. In principle, neuroinflammation plays a protective role by limiting tissue damage and restoring homeostasis through the containment and resolution of pathological insults. However, sustained neuroinflammatory activation shifts its function from protective to detrimental, underpinning progressive cellular damage, driving disease progression [146], and leading to glymphatic dysfunction. Astrocyte activation and AQP4 depolarization represent the most direct mechanisms, which have been elaborated in detail above. The subsequent analysis will focus on exploring their relationship from alternative perspectives.

As the primary drivers of the inflammatory response, microglia undergo extensive proliferation during neuroinflammation. They release pro-inflammatory cytokines like IL-1β and TNF-α, which directly act on astrocytes to induce the formation of reactive A1 subtype [147], prompting the aforementioned AQP4 depolarization and remodeling of the end-foot structure [148]. What’s more, microglia recruit peripheral immune cells to the CNS and its PVS by releasing chemokines such as CCL2 and CXCL10 [149, 150]. Successive waves of these infiltrating cells accumulate progressively, creating a physical barrier that obstructs CSF inflow and outflow [151]. In the advanced stages, neuroinflammation causes a cascade of irreversible damage, including disruption of the BBB and neuronal loss, which ultimately destroys the structural integrity of the glymphatic system.

Interestingly, neuroinflammation can be remotely induced by peripheral injury or inflammation, which appears to be related to the trafficking of circulating immune cells. For example, CD4^+^ T cells originating from the paracolic lymph nodes could migrate to the meninges, where they activate the NLRP3 inflammasome [152]; Circulating monocytes can accumulate in the PVS and differentiate into PVMs, impeding glymphatic flow [153]. The central and peripheral immune systems are not isolated entities but instead engage in dynamic crosstalk. Future research should prioritize elucidating the mechanisms and functional consequences of this central–peripheral immune interaction.

### Glymphatic circulation dysfunction

The glymphatic flow begins with CSF entering the periarterial spaces from the subarachnoid space. After mixing with ISF in the brain parenchyma, the fluid is ultimately cleared along perivenous pathway into the peripheral lymphatic system. In brief, the glymphatic circulation can be conceptualized as three distinct yet functionally interconnected stages: inflow, exchange, and clearance. The inflow and clearance processes are primarily driven by pulsatile forces from the vessel wall and are hydrodynamically modulated by the physical resistance within PVS. For example, cerebral edema and elevated intracranial pressure induced by traumatic brain injury (TBI) cause brain tissue swelling, which reduces PVS volume and consequently increases resistance to glymphatic flow. These observations are supported by imaging data demonstrating impaired clearance of intrathecal contrast agents and diminished transport to the deep cervical lymph nodes [154]. The clearance of metabolic waste through the CSF-ISF exchange critically depends on AQP4 protein channels polarized at the end-feet of astrocytes. Studies have demonstrated that the loss of AQP4 localization around perivascular was associated with Aβ and p-tau deposition in post-mortem brain tissue from patients with AD [155].

Glymphatic impairment in neurological disorders typically encompasses multifactorial processes, driven primarily by aberrant crosstalk among structural, functional, and molecular-metabolic factors, rather than arising from a single isolated event. These factors act synergistically, establishing a self-perpetuating vicious cycle that couples neurological disease progression with glymphatic dysfunction. In this section, we will discuss the mechanisms of glymphatic impairment in several common neurological disorders, as shown in Table 3.

**Table 3:** The mechanisms of glymphatic impairment in neurological disorders

Neurological disorders	Impaired step	Mechanisms	Outcomes	Reference
TBI	Glymphatic drainage and clearance	Norepinephrine storm reduced the contractility of cervical lymphatic vessels	Leading to post-traumatic cerebral edema	[156]
MMD	Glymphatic flow and clearance	Enlarged lateral ventricles and arterial stenosis impaired cerebral hemodynamics	Poor prognosis and an increased risk of hemorrhage	[157]
AD	CSF-ISF exchange	The loss of AQP4 polarization impaired clearance of Aβ and tau	Cognitive dysfunction was exacerbated	[158]
Stroke	Glymphatic flow	Glial-vascular unit decoupling weakened the driving force of glymphatic circulation	Developing post-stroke depression	[159]
MS	Glymphatic inflow	The enlargement of the CP impaired cerebrospinal fluid production	Triggering dysregulated neuroinflammatory responses, increasing the risk of clinical disability	[160]
Narcolepsy	Impaired CSF dynamics	Sleep–wake rhythm disruption gives rise to regular norepinephrine oscillations that cause damage	Exacerbating disease severity	[161]
SE	Glymphatic clearance	Dysfunction of the glial–vascular unit and neuroinflammation impair glymphatic flow	Acute cerebral edema and long-term cognitive impairment	[162]
ICH	Glymphatic inflow	Brain damage disrupts the normal structure and function of the glymphatic system	Post-hemorrhagic brain edema and cognitive impairment	[163]

TBI, Traumatic brain injury; MMD, Moyamoya disease; AD, Alzheimer’s disease; AQP4, Aquaporin 4; Aβ, Amyloid beta; CSF, Cerebrospinal fluid; ISF, Interstitial fluid; MS, Multiple sclerosis; CP, Choroid plexus; SE, Status epilepticus; ICH, Intracerebral hemorrhage

### Sleep disorders and circadian dysregulations

Sleep disorders are clinical syndromes characterized by abnormalities in sleep initiation, maintenance, architecture, duration, or quality [162], leading to daytime impairments including fatigue, poor concentration, and mood disturbances. Circadian dysregulations involve pathological misalignment between an individual's internal circadian pacemaker and the 24-h environmental light–dark cycle [163], resulting in persistent and maladaptive shifts in sleep onset and offset. As established, the sleep–wake cycle and circadian rhythm govern glymphatic flow. Specifically, during NREM stage N3 sleep, synchronized slow-wave activity [164], systemic vasomotion, and respiratory pulsations [165] converge to amplify fluid transport. This physiological synchrony maintains circadian AQP4 polarization [166], optimizing glymphatic clearance and preserving cerebral proteostasis.

However, sleep disorders and circadian dysregulations have reached epidemic proportions in modern society. Epidemiological data indicate that chronic sleep deprivation, social jet lag, and frequent sleep disturbances are highly prevalent among adults, with 29.8% experiencing trouble sleeping and 27.2% suffering from daytime sleepiness [167]. This trend can be attributed to several contemporary factors, including high-pressure work occupational environments, prolonged screen time, chronic psychological stress, and the widespread consumption of neuro-stimulants such as caffeine and sleep-disrupting substances like alcohol [168]. Consequently, future management should be grounded in the bio-psycho-social medical model, which simultaneous monitoring of a patient's biological markers, subjective well-being, and social functioning within a unified follow-up record. The overarching therapeutic objective is to ensure the coordinated progression of these three domains.

## Glymphatic dysfunction in neurological disorders

**Fig. 4:**
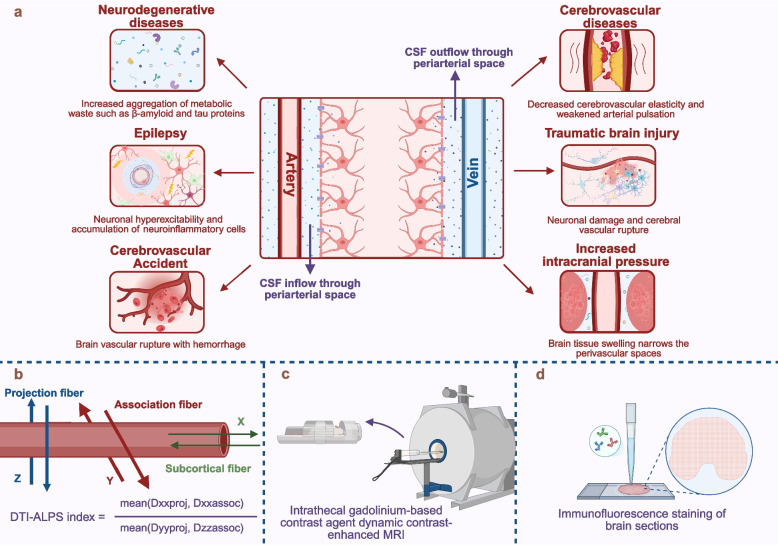
**a** Key mechanisms contributing to glymphatic dysfunction in neurological disorders. Techniques for assessing glymphatic circulation, including (**b**) diffusion tensor image analysis along the perivascular space (DTI-ALPS), (**c**) intrathecal gadolinium-based contrast agent dynamic contrast-enhanced magnetic resonance imaging (MRI), and (**d**) fluorescently labeled perfusion-fixed coronal sections from adult mice

Clinical studies have found that glymphatic dysfunction is prevalent across various neurological diseases. Targeted therapeutic strategies, including cervical lymph node-to-vein anastomosis [169] which could expand cerebral glymphatic outflow pathways and improve cognitive function in patients with AD in the short term, however, its long-term effects are suboptimal. A key contributing factor is that the dynamic factors influencing glymphatic circulation, particularly the rate of glymphatic flow, have not been sufficiently optimized. Investigating these dynamic mechanisms is crucial for developing novel approaches against neurological diseases (Fig. 4). In the sections that follow, we provide a distinct analysis categorized by neurological disease subtype, as outlined in Table 4.

**Table 4:** Comparison of the mechanisms underlying glymphatic dysfunction across various neurological diseases

Disease	Common Mechanisms	Unique Mechanisms
Neurodegenerative diseases	AQP4 aquaporin polarity loss: Reduced expression or depolarized of AQP4 decreases CSF-ISF exchange Neuroinflammation and astrogliosis: Microglial activation and other inflammatory responses impair astrocytic and vascular function Reduced circulatory driving force: Accumulated metabolites slow down CSF flow	Accumulation of specific toxic proteins: Accumulation of toxic proteins such as Aβ, α-syn, and TDP-43 Interacting with aging and sleep disorders: Forming a bidirectional vicious cycle with aging and sleep disorders
Cerebrovascular diseases	Destruction of the BBB: Impairing the architecture of glymphatic system AQP4 aquaporin polarity loss: Ischemia and hypoxia directly injure astrocytes, impairing both the function and expression of AQP4 Reduced circulatory driving force: Vascular injury attenuates arterial pulsation	Sudden vascular occlusion or rupture acutely compromises local glymphatic flow. The ensuing neuroinflammation and edema then compress the perivascular spaces Circulatory blockage: Blood-clot components form physical emboli within PVS Vascular structural damage: Chronic cerebrovascular disease frequently manifests as thickened and stiffened vessel walls, which diminish the driving force
Traumatic brain injury	Neuroinflammation and astrogliosis: Post-injury acute neuroinflammation is triggered AQP4 aquaporin polarity loss: Astrocytes shift to a toxic phenotype, leading to reduced AQP4 polarization	Mechanical injury: Directly causing destruction of blood vessels and brain tissue Acute and delayed sequelae: Glymphatic dysfunction is associated with both acute cerebral edema and chronic cognitive impairment
Multiple sclerosis and Neuroinflammation	Neuroinflammation: Persistent neuroinflammation driven by central and peripheral immune cells AQP4 aquaporin polarity loss: Astrocytes shift to a toxic phenotype, leading to reduced AQP4 polarization Metabolic waste accumulation: Massive accumulation of inflammatory metabolic waste	Perivenous and meningeal neuroinflammation directly disrupt the glymphatic circulation Autoimmune attack: Some neuroinflammatory diseases produce autoantibodies against AQP4
Epilepsy	Destruction of the BBB: Impairing the architecture of glymphatic system Neuroinflammation: Seizures trigger a neuroinflammatory storm Metabolic waste accumulation: Massive accumulation of inflammatory metabolic waste	Neural network disruption: Abnormal neuronal firing loses the rhythmic drive on CSF
Neurodevelopmental and Psychiatric Disorders	Neuroinflammation: Persistent low-grade neuroinflammation AQP4 aquaporin polarity loss: Astrocytes shift to a toxic phenotype, leading to reduced AQP4 polarization	Sleep rhythm disruption: Forming a bidirectional vicious cycle with sleep disorders Mostly occurs in early neurodevelopment and is strongly influenced by environmental and genetic factors

AQP4, Aquaporin 4; Aβ, amyloid-beta; α-syn, α-synuclein; TDP-43, TAR-DNA-binding protein 43; CSF, Cerebrospinal fluid; BBB, Blood–brain barrier; PVS Perivascular spaces

### Neurodegenerative diseases

Neurodegenerative diseases (NDs) are a group of diseases marked by the progressive neuronal loss in the central or peripheral nervous system, often accompanied by the accumulation of misfolded proteins, including Aβ, α-syn, TAR-DNA-binding protein 43 (TDP-43), and mutant huntingtin protein [170–173]. Early investigations into protein degradation within the CNS mainly focused on intracellular mechanisms like the proteasomal and lysosomal pathways. However, recent studies have revealed that the extracellular clearance pathways, such as those mediated by CSF and ISF, are equally important. For instance, in Huntington’s disease (HD) mice, impaired glymphatic influx and clearance precede striatal atrophy and motor deficit [174]. Failure to clear pathological huntingtin proteins permits their prion-like propagation along CSF-ISF pathways, directly exposing neurons to toxic aggregates [175]. Therefore, glymphatic dysfunction may be implicated early on and exert a persistent driving role in the pathogenesis of in NDs.

Beyond impaired protein clearance, glymphatic dysfunction in NDs encompasses several other shared pathological mechanisms. Aging is a major risk factor for NDs, the most prevalent forms of which occur primarily in the elderly. While aging is associated with the narrowing and rarefaction of meningeal lymphatic vessels (mLVs) [176]. Additionally, comorbidities including atherosclerosis, hypertension, and diabetes diminish arterial wall compliance, weakening the pulsatile driving force that supports glymphatic flow. Disruptions in circadian rhythms and sleep–wake cycles are common in NDs [177]. On the one hand, proteostatic dysfunction in NDs impairs neurons that regulate sleep–wake cycles, resulting in deficits in slow-wave and NREM sleep [178]. On the other hand, sleep deprivation and circadian rhythm disturbances accelerate disease progression by hampering key clearance mechanisms, including proteasomal degradation, autophagy, the unfolded protein response, and glymphatic function [179]. What’s more, the NDs-induced astrocytic phenotypic switching [180], coupled with the ensuing chronic low-grade neuroinflammation [181], are also key contributing factors.

### Cerebrovascular diseases

Cerebrovascular diseases are characterized by structural or functional changes in cerebral vasculature, mainly including cerebral ischemia, hemorrhage, and impaired brain function. It has been reported that cerebrovascular pathologies disrupt extracellular matrix–related genes in endothelial, mural, and fibroblast cells [182], which form the critical anatomical foundation of the glymphatic system. In this section, we will outline the underlying mechanisms stratified by the acuity of disease onset.

Stroke is the most common acute cerebrovascular disease, comprising two main subtypes: ischemic stroke (IS) and hemorrhagic stroke (HS). IS arises from occlusion of cerebral blood flow, which directly impairs the rhythmic pulsatility of cerebral vessels. Rodent models of acute ischemic stroke demonstrate damaged paravascular CSF influx and Aβ deposition within the PVS after middle cerebral artery occlusion (MCAO), as evidenced by MRI and histological examination [115, 183]. Strikingly, pharmacological enhancement of glymphatic function alleviated brain edema after IS, accompanied by reduced Aβ plaque burden and improved cognitive performance [184]. HS is a highly devastating stroke subtype associated with substantial morbidity and mortality. It undermines the structural integrity of the BBB, allowing plasma components to leak into the brain parenchyma and disrupting the critical pressure gradient required for CSF and ISF exchange. Additionally, blood components, especially fibrin and fibrinogen deposits, form obstructive physical casts within the PVS, mechanically obstructing glymphatic circulation.

Chronic cerebrovascular disease (CCVD) is a clinical syndrome resulting from the long-term cumulative effects of various etiologies, which is characterized by chronic cerebral hypoperfusion and small vessel pathology. Among most patients aged older than 70 years with CCVD, post-mortem examinations consistently reveal small-vessel sclerosis and irregular, tortuous dilatation [185, 186], driven by chronic perivascular inflammation and sustained tissue hypoxia. Imaging demonstrates characteristically enlarged perivascular spaces (EPVS) [187]. It should be emphasized that EPVS do not indicate increased glymphatic clearance. Instead, they represent a passive consequence of perivascular efflux, manifesting as physical stasis and compensatory dilatation secondary to vascular wall stiffening and channel obstruction [188, 189].

### Traumatic brain injury

Traumatic brain injury (TBI) refers to structural and physiological brain damage induced by external mechanical forces. Current treatments for TBI are still focus on symptom-based management, including craniotomy, oxygen therapy, and hypothermia support, which serve as adjunctive measures [190]. However, conventional therapeutic strategies yield limited efficacy, which fail to reliably prevent complications such as cerebral edema or post-traumatic epilepsy, and they may leave survivors with a persistent, elevated lifelong risk of NDs [191].

Recent evidence suggests that TBI reduces glymphatic influx and hinders the clearance of intracortically injected radiotracers from the ISF [192], which are associated with the non-uniform changes in expression and distribution of AQP4 across different cortical layers [193]. Therefore, glymphatic system may offer novel insights into the treatment and recovery of TBI, which has already been robustly verified. For example, angiotensin II type 2 receptor (AT2R) activation restored perivascular AQP4 polarization and restored cerebral blood flow, promoting neurological recovery after TBI [194]; Activation of the glucagon-like peptide-1 receptor (GLP-1R) restored glymphatic transport following TBI, improving cognitive function [195]. Nevertheless, the bulk of existing evidence is derived predominantly from preclinical animal models, and successful clinical translation of these findings remains to be fully validated.

### Multiple sclerosis and neuroinflammation

Multiple sclerosis (MS) is a chronic immune-mediated disorder of the CNS, characterized by demyelination, axonal damage, and neuroinflammation. Chronic neuroinflammation not only represents a core pathological hallmark of MS but also acts as a fundamental driver of disease progression. In relapsing–remitting MS (RRMS), neuroinflammation is primarily driven by the dysregulated interaction among peripheral T cells, B cells, myeloid cells, and their effector and regulatory subsets [196]. In secondary-progressive MS (SPMS), this inflammation transitions to a chronic, diffuse, and self-sustaining process driven by the resident innate immune cells of CNS [197], which burns out the axons and lead to neuronal death. Moreover, inflammation accumulates around the veins and PVS, forming perivascular cuffs that appear on imaging as the right-angle demyelination sign or Dawson's finger [198]. Another important site of involvement is the leptomeninges [199], where innate and adaptive immune cells can organize into tertiary lymphoid structures, propagating smoldering inflammation. Damage to the leptomeningeal disrupts the shunting process of CSF from periarterial to perivenous spaces [161], while venous involvement directly impairs CSF outflow pathways. Meanwhile, imaging studies have also demonstrated a globally reduced ALPS index in patients with MS, which correlates with the severity of demyelination and neurodegeneration [27]. Consequently, MS impairs the glymphatic circulation, and this impairment in turn accelerates the disease progression. Targeting the glymphatic system may therefore provide a novel strategy for the diagnosis and management of MS.

### Epilepsy

Epilepsy is a collection of neurological disorders characterized by abnormal neuronal discharges, resulting in spontaneous and recurrent unprovoked seizures. Preclinical studies have shown that acute seizures impair glymphatic function by disrupting the supportive role of high-energy ionic waves generated by synchronized action potentials in CSF circulation [162], as well as by inducing subcellular mislocalization of AQP4 [200]. Similarly, glymphatic system function can be restored after the resection of the epileptogenic focus [23]. At the same time, the dysfunction of glymphatic circulation may also contribute to the pathogenesis of chronic epilepsy and its associated complications. Evidence indicates that a reduced ALPS index correlates with seizure severity, as well as the degree of cognitive impairment and depressive symptoms [165, 201]. However, most existing studies reporting such associations are limited by small sample sizes. Future work is warranted to establish causal relationships and validate consistency across larger cohorts.

### Neurodevelopmental and psychiatric disorders

Neurodevelopmental disorders (NDDs) generally emerge during early brain development and are marked by deficits in personal, social, academic, and occupational functioning [166]. Given that inflammation and the dysregulation of specific neurotransmitters are two influential pathogenic mechanisms underlying NDDs, the role of glymphatic system in these disorders has gained increasing recognition. Enlarged perivascular spaces and a reduced ALPS index have been observed in individuals with attention-deficit/hyperactivity disorder [167], pediatric tourette syndrome [168], and autism spectrum disorder [169].

Psychiatric disorders are characterized by significant disruptions in thought, emotion, and behavior, often resulting from the complex interplay of genetic predisposition, environmental stressors, and traumatic experiences. Recent studies have identified that glymphatic circulation is impaired in psychiatric disorders [170, 202, 203]. A common denominator across these conditions appears to be sleep disturbance. For example, patients with depression often experience difficulty falling asleep and early morning awakening [204]; Bipolar disorder is frequently associated with disruptions in circadian rhythms [205]. Therefore, therapeutic targeting of sleep–wake architecture to enhance glymphatic clearance presents a promising adjunctive strategy for psychiatric disorders.

## Experimental and imaging approaches

In the following section, we provide a comprehensive review of widely used detection approaches in preclinical animal and clinical human studies, along with a focused discussion on the key challenges in translating preclinical findings into clinical practice.

### Animal research

Animal research technique has been well-established, including key molecular assays, CSF kinetic analyses, and targeted modulation strategies. Subsequently, we will outline the most prevalent methodologies in this filed.

#### Molecular markers and quantification of AQP4

AQP4 is the central regulator of glymphatic function and is the most widely used readout in animal studies, which can be quantified through Western blotting and visualized using immunofluorescence labeling. According to the western blot analysis, AQP4 polarization was quantified by the M23-to-M1 isoform ratio [175]. In immunofluorescence imaging, this polarization is assessed by calculating the ratio of AQP4 signal within a perivascular annulus to the global AQP4 signal [176]. Consequently, pharmacological modulation and genetic manipulation of AQP4 are also vital approaches to elucidate AQP4-dependent mechanisms.

#### Assessment of MLVs

MLVs is the primary outflow pathway for clearing metabolic waste from the brain to the cervical lymph nodes. They express transcription factors and cell surface markers characteristic of lymphatic endothelium [177], such as lymphatic vessel endothelial hyaluronan receptor-1 (LYVE-1) and Prospero homeobox protein 1 (PROX1). The fluorescence staining of these markers can indirectly evaluate the development and function of MLVs. Conventional imaging techniques are limited in distinguishing the structure and function of MLVs from glymphatic-circulatory network. Importantly, Fei Yang and colleagues utilized dual-contrast functional photoacoustic microscopy (DCF-PAM) to achieve the first in vivo co-localization imaging of MLVs and cerebral vasculature [206], enabling high-resolution 3D visualization within the meninges.

#### Tracer tracking

Tracer tracking enables real-time monitoring of the entire process of CSF influx, distribution, and clearance. When available, dynamic contrast-enhanced MRI (DCE-MRI) is the gold-standard approach for evaluating glymphatic function in live animal models, as it is the only method that provides comprehensive visualization and spatiotemporal characterization of the entire system [179]. However, its application is constrained by stringent technical and infrastructural demands. A more common alternative involves euthanizing animals at sequential time points after tracer injection, followed by fluorescence microscopic analysis of tracer distribution in brain tissue and deep cervical lymph nodes Sects. [207]. Nonetheless, the aforementioned approaches fail to directly evaluate brain-wide three-dimensional (3D) CSF flow. The recently developed fluorescence micro-optical sectioning tomography (fMOST) combines automated serial sectioning with high-resolution optical imaging, enabling the reconstruction of 3D atlases of the glymphatic pathway [208].

#### in Vivo Two-photon microscopy imaging

The glymphatic system was firstly identified via in vivo two-photon microscopy imaging (2PM), which employs near-infrared lasers to excite fluorescent tracers, enabling the direct visualization of CSF inflow pathways and its driving mechanisms [10]. Nevertheless, 2PM typically requires cranial window surgery and is restricted to superficial cortical layers, hindering the visualization of deep brain structures [182]. Therefore, it is often combined with ex vivo slice imaging or magnetic resonance imaging for complementary analysis.

#### Deep cervical lymph node post-ligation

Waste fluid cleared by the glymphatic system is transported through MLVs and drains into the cervical lymph nodes, primarily the deep cervical lymph node (dcLN) [209]. Surgical ligation of the dcLN can artificially obstruct glymphatic drainage and is employed as an experimental tool in mechanistic studies. For example, dcLN ligation has been reported to exacerbate AD-like pathology, resulting in increased neuroinflammation, synaptic protein loss, and deficits in cognitive and exploratory behaviors [184].

### Human research

Clinical imaging of the glymphatic pathway is an emerging field, with several innovative methods recently developed to visualize CSF flow in human brain. Various MRI-based approaches have been developed, each permitting characterization of distinct aspects of glymphatic and perivascular physiology.

#### Dynamic contrast-enhanced MRI

Contrast-enhanced MRI using intrathecal gadolinium-based contrast agents (GBCAs) allows for the dynamic tracking of CSF circulation pathways, similar to tracer-based techniques in animal studies [210]. However, intrathecal injection is regarded as an invasive procedure in clinical practice and has not been approved as a standard administration route. To address these concerns, intravenous injection of GBCAs has been explored, which permits the contrast agents to enter the glymphatic circulation through the dura mater, choroid plexus, and several other brain regions [186]. This method is the only FDA-approved contrast-enhanced MRI technique and is routinely employed in clinical practice. For example, serial T1-weighted 3 T MRI was used to quantify GBCA clearance kinetics, in combination with CSF proteomics [187], to identify protein biomarkers closely associated with glymphatic dysfunction in patients with amnestic mild-to-moderate cognitive impairment.

#### DTI-ALPS

DTI-ALPS measures the diffusivity along PVS within major white matter tracts by calculating the anisotropy ratio of water molecule diffusion in these regions adjacent to white matter fiber tracts at the level of the lateral ventricles [188, 189]. Due to its non-invasive nature and scalability for large cohorts, it has become the most widely used technique for clinical assessments. Consistently, reduced ALPS values have been documented across a range of neurological disorders. However, it should be emphasized that DTI-ALPS is an indirect measure reflecting global glymphatic clearance function averaged across an entire cerebral hemisphere. It lacks the spatial resolution needed to resolve local or region-specific variations in clearance activity. Furthermore, the ALPS index inherently conflates perivascular diffusion with axonal integrity, which may lead to overestimation of glymphatic-specific changes [190].

#### EPVS

The PVS constitute the primary structural channel of the glymphatic system. Cumulative tissue injury leads to structural remodeling within the glymphatic system, which are predominantly observed as EPVS on MRI and can serve as an additional marker for assessing glymphatic function [191]. For instance, the ALPS index and EPVS burden were evaluated in combination to investigate the effects of REM sleep behavior disorder (RBD) on glymphatic function in patients with PD [192].

#### BOLD-CSF coupling

The blood-oxygen-level-dependent (BOLD) signal is a functional magnetic resonance imaging (fMRI) measure that indirectly reflects brain functional connectivity through local changes in blood oxygenation caused by neuronal activity [193]. Studies have indicated that the global BOLD signal is closely linked to specific electrophysiological events lasting 10 to 20 seconds [194], which correspond to transient arousal modulation during drowsiness or sleep. Similarly, a study demonstrated a direct coupling between CSF fluctuations in the fourth ventricle and low-frequency hemodynamic oscillations in the human cortex, which can be interpreted as an indication of increased brain clearance activity [195, 211]. Therefore, the BOLD-CSF coupling captures cerebral hemodynamic changes and spinal CSF inflow fluctuations into the brainstem simultaneously [197]. This allows its use as a non-invasive imaging marker for assessing the functional status of the glymphatic circulation, where stronger coupling indicative of more efficient CSF flow.

#### Arterial spin labeling MRI

Arterial spin labeling (ASL) MRI employs magnetically labeled arterial blood water as an endogenous tracer to quantify its transport across the blood-cerebrospinal fluid barrier (BCSFB) within the ventricular choroid plexus [198]. It permits noninvasive visualization of glymphatic fluid exchange efficiency. For example, Caihua Ye and colleagues utilized ASL imaging to evaluate whether intranasal oxytocin can enhance intracranial glymphatic drainage in AD mouse models [212].

### Challenges and limitations in translating animal findings to humans

Despite extensive research efforts, many aspects of the glymphatic system remain poorly understood, and translational transition from animal models to human clinical practice continues to be a formidable hurdle. This knowledge gap is largely attributable to the lack of a standardized, non-invasive methodology capable of quantifying glymphatic function in clinical research. Animal studies frequently employ 2PM and tracer tracking to directly visualize CSF flow dynamics in real time. While tracer tracking is also utilized in human studies, it typically yields indirect data with low spatial resolution [213], which complicates the accurate reproduction of the precise glymphatic pathways observed in mouse models. Although multimodal MRI can improve assessment accuracy, it also prolongs scan duration and increases costs, imposing an additional burden that may be untenable for some patients. Canonical glymphatic biomarkers, such as AQP4 [214] and Lyve-1 [215], are primarily sequestered within cellular membranes or restricted intracellular compartments, yielding negligible concentrations in accessible biofluids including CSF and plasma. This renders their precise in vivo quantification currently unfeasible in humans. To circumvent these constraints, future research should prioritize high-resolution neuroimaging with enhanced molecular specificity, along with multimodal data integration to capture their dynamic features.

Additionally, non-negligible interspecies differences exist in the physiological and anatomical features of the glymphatic system. For example, the human brain's complex cortical folding contrasts with the lissencephalic murine brain [216], which possesses a comparatively simplified glymphatic network. More importantly, AQP4 displays distinct polarization profiles between the mouse and human brain [217]. In the mouse brain, AQP4 exhibits far stronger polarized enrichment at perivascular astrocytic end-feet, with ratios of 5:1 to 10:1 in mice, as opposed to 2:1 to 3:1 in humans. This differential polarization suggests that experimental phenotypes reliant on AQP4 are more pronounced in mice. Future work ought to prioritize the identification of animal models and brain organoids that more accurately replicate human brain anatomy and functional phenotypes.

The challenge of reproducing causal relationships represents another critical challenge. In animal experiments, causal links between the glymphatic system and neurological diseases can be clearly established through pharmacological or gene-targeted interventions. In contrast, most human studies are restricted to identifying correlations, as ethical and practical limitations preclude analogous experimental interventions. Large-scale, multicenter cross-sectional and longitudinal studies, paired with assessments of minimally invasive interventions such as focused ultrasound, provide a promising avenue for establishing causality in human glymphatic research.

## Potential therapeutic approaches

Therapeutic strategies aim at enhancing glymphatic flow, together with pharmacotherapies directly targeting the glymphatic system, represent innovative approaches in neurobiology. Guided by the functional properties and underlying regulatory mechanisms of glymphatic circulation, we identified and summarized several promising therapeutic strategies.

### Pharmacological modulation of AQP4 and astrocytic function

The polarized localization of AQP4 on astrocytic end-feet is indispensable for glymphatic function, making it a highly attractive therapeutic target. Table 1 summarized interventions targeting AQP4 expression and polarity in animal experiments. Despite successful elucidation of these mechanisms in animal models, these findings have not yet been translated into clinical practice. In contrast, pharmacological strategies aimed at modulating the polarized phenotype of astrocytes are more clinically tractable. For example, Glucagon-like peptide-1 (GLP-1) is an incretin hormone derived from the gut, primarily secreted by L cells in the intestine in response to food intake [218]. In addition to its function in glucose homeostasis, GLP-1 also modulates cell survival and death [219]. Furthermore, pathological α-syn has been shown to induce microglial activation and upregulate the expression of microglial GLP-1 receptors in PD models [220]. NLY01, an engineered exendin-4 and GLP-1 receptor (GLP-1R) agonist, selectively suppresses microglial activation via GLP-1R and limits reactive astrogliosis in PD [221] and AD models [222]. Encouragingly, the clinical application of NLY01 for PD has progressed to Phase II trials, which have successfully confirmed its safety, tolerability, and potential motor benefits in younger patient populations [223]. However, its potential to enhance glymphatic circulation awaits rigorous experimental and clinical validation. Additionally, oral drugs typically exhibit a low intracranial transfer efficiency and limited brain penetrance, future investigations should prioritize alternative delivery routes such as intranasal or intrathecal administration and advanced drug-delivery systems such as nanocarriers, hydrogels, to improve intracranial transport efficiency.

### Enhancing vascular pulsation and CSF circulation

Once the accumulation of metabolic waste in the CSF reaches a critical threshold, it initiates a self-reinforcing cycle of glymphatic dysfunction. At this pathological stage, interventions designed to enlarge outflow pathways or enhance CSF–ISF exchange offer limited benefits. By contrast, strategies that enhance the driving force of circulation and increase CSF flow velocity demonstrate greater efficacy.

The regular pulsation of blood vessels serves as the primary driving force for glymphatic circulation. Aging and comorbidities such as hypertension and hyperlipidemia, which trigger cerebrovascular stenosis and arteriosclerotic remodeling, attenuate this pulsatile propulsion. Accordingly, interventions targeting these underlying vascular pathologies provide a vital therapeutic strategy. For instance, antihypertensive agents could maintain blood pressure within an optimal range, preventing microvascular shear stress caused by hypertension or insufficient supply due to hypotension [224]. Management of serum lipid levels stabilizes arterial plaques, preserves vascular patency and elasticity [225], and facilitates the restoration of the driving force for CSF circulation. Moreover, drugs like sildenafil could improve cerebral vascular function through the selective inhibition of phosphodiesterase type 5 (PDE5) activity in vascular endothelial cells [226]. These compounds are progressively being evaluated in clinical trials for neurological disorders [227, 228], and may represent promising pharmacological agents for enhancing glymphatic circulation.

Recent studies also provide promising evidence supporting the efficacy of some physical modulation techniques. Transcranial focused ultrasound (tFUS), with its superior spatial resolution and deep targeting capabilities, has emerged as a next-generation neuro-modulatory modality [229]. Recent evidence indicates that FUS improved CSF circulation efficiency and facilitated solute exchange between CSF and ISF [230]. This beneficial effect is likely mediated by acoustic energy-driven diffusion and convection of CSF [231], as well as ultrasound-induced peristaltic deformation of blood vessels [232]. For example, animal studies have confirmed that focal low-intensity focused ultrasound (LIFU) stimulation of the unilateral M1 cortex in mice induces rapid CSF inflow and restores glymphatic circulation in both hemispheres following a photothrombotic stroke [233]; FUS has been demonstrated to enhance the clearance of Aβ in AD mouse models [234]. However, its potential for clinical translation remains in the experimental phase. A first-in-human Phase I trial has evaluated the safety and reproducibility of FUS technology in patients with AD [235], but its ability to enhance glymphatic clearance remains to be determined. Both 40 Hz multisensory gamma stimulation and repetitive transcranial magnetic stimulation (rTMS) demonstrate comparable therapeutic potential. Mechanistically, 40 Hz gamma stimulation is proposed to enhance glymphatic function by promoting the polarized distribution of AQP4 and intensifying vascular pulsatility through adenosine A2A receptor (A2AR) signaling [236]. However, clinical evidence is currently insufficient. rTMS is an FDA-approved non-pharmacological modality with a clinical footprint spanning over 15 years in the management of neuropsychiatric disorders such as depression and obsessive–compulsive disorder [237, 238]. Some studies suggested that rTMS may improve glymphatic circulation [239], but this claim currently lacks robust supporting evidence.

Additionally, we would like to emphasize the translational potential of brain-computer interface (BCI) system. As discussed in the previous mechanistic section, glymphatic dysfunction is fundamentally multifaceted, encompassing disrupted sleep architecture, aberrant respiratory rhythms, impaired regulation of vascular tone, and autonomic nervous system dysregulation. All of these factors could trace to dysregulated activity within discrete neural nuclei. BCI systems create a direct communication link between the brain and external devices, translating abnormal neural signals into commands for precise neuromodulation [240]. Theoretically, it could detect aberrant signals indicative of glymphatic dysfunction and initiate targeted electrical or pharmaceutical interventions to regulate the physiological processes governing glymphatic function. However, the relevant neural oscillations are often too weak to be detected by existing BCI electrode platforms. Given that glymphatic flow is also driven by hemodynamic forces, monitoring neuro-electrophysiological signals that regulate blood flow provides a viable alternative for indirect assessment, which is the focus of our current research. For instance, non-invasive fMRI detects neuronal activity by recording BOLD signals [241], whereas functional near-infrared spectroscopy (fNIRS) enables direct measurement of cortical hemoglobin oxygenation to characterize local cerebral hemodynamics [242], facilitating the development of blood-flow-driven BCI systems. Furthermore, implantable intravascular BCI electrodes that decode real-time hemodynamic signals, such as cerebral blood flow velocity and volume, represent a promising alternative. Nevertheless, these strategies remain largely theoretical at present, and the establishment of a fully integrated bidirectional regulatory loop still warrants extensive further investigation.

### Anti-inflammatory and antioxidative interventions

Neuroinflammation and oxidative stress are the core pathogenic drivers of glymphatic circulation impairment. Most neurological diseases are accompanied by acute or chronic neuroinflammation, characterized by microglial and astrocytic activation, as well as the BBB disruption. These sequential pathological processes disrupt multiple facets of glymphatic circulation, as extensively elaborated in the dedicated section outlining neuroinflammation mechanisms. However, current research on anti-inflammation and antioxidative stress to enhance glymphatic circulation is largely confined to animal models. In contemporary clinical practice, potent anti-inflammatory and antioxidative therapies are predominantly reserved for CNS pathologies with definitive autoimmune or infectious etiologies [243, 244], such as MS, neuromyelitis optica spectrum disorder, autoimmune encephalitis, and CNS infections. For most non-inflammatory neurological conditions, clinical management remains largely supportive and symptom-focused. Surprisingly, the clinical efficacy of several anti-inflammatory agents, such as omega-3 fatty acids [245] and NT-0796 [246], are currently under evaluation. We believe that anti-inflammatory and antioxidant therapies will evolve into routine adjunctive treatments in future clinical practice.

### Lifestyle and physiological approaches

In addition to pharmacological and physical interventions, lifestyle modifications and physiological regulation are equally important. The clearance rate of the glymphatic system during sleep is significantly higher than in the awake state [247]. However, sleep disorders have become a concern in modern society, driven largely by psychological stress stemming from the demands of multiple social roles and chronic overwork [248]. These pressures, rooted in sociocultural and economic drivers, put the brain in a persistent state of heightened arousal, markedly reducing sleep duration and elevating the prevalence of insomnia. Additionally, unhealthy lifestyle habits, such as prolonged use of electronic devices before bed, consumption of stimulants like caffeine, alcohol, and nicotine, and irregular sleep schedules, disrupt the endogenous circadian rhythmicity [249, 250]. Consequently, effective interventions must extend beyond sleep alone to encompass core pillars of health, including stress regulation, emotional well-being, and stable daily routines. When sleep disorders stem from underlying medical conditions or the physiological changes of aging, pharmacological intervention with sedative-hypnotic medications may be warranted. Optimal pharmacotherapies should aim to achieve more than merely extending sleep duration. For example, while zolpidem effectively prolongs sleep duration, it also suppresses essential nighttime norepinephrine oscillations [251], which adversely affect glymphatic function. By contrast, melatonin has been demonstrated to enhance glymphatic function through its fundamental role in circadian regulation [252]. However, direct clinical evidence supporting the use of sleep aids to enhance glymphatic flow remains lacking, highlighting the necessity for further research.

Regular voluntary exercise has emerged as a potent non-pharmacological intervention for enhancing glymphatic circulation via the upregulation and polarization of AQP4 [253], and improved neurovascular coupling [254]. A healthy diet rich in omega-3 polyunsaturated fatty acids and low in salt, sugar, and ultra-processed foods may reduce neuroinflammation [255], and thereby promote the recovery of glymphatic function. Moreover, psychological and emotional regulation strategies, including meditation, deep breathing, and music therapy, can alleviate glymphatic impairment caused by sustained elevations in cortisol levels [256].

## Conclusion and future perspectives

As the primary clearance infrastructure of brain, glymphatic system is critical in waste removal and metabolite homeostasis, providing transformative insights into the pathophysiological mechanisms and clinical management of neurological disorders. Emerging researches indicates that glymphatic dysfunction even manifests prior to symptomatic onset in certain neurological disorders [257]. Specifically, a reduced ALPS index predicts conversion to amyloid-positive status, an increased likelihood of clinical progression, and accelerated cognitive decline [258]. Extending beyond neurodegenerative pathologies, evidence in patients with episodic migraine indicates that a reduced DTI-ALPS index correlates with increased attack frequency [259]. This suggests that impaired waste clearance may contribute to the chronification of pain. Consequently, glymphatic dysfunction may serve as a sensitive biomarker for the early detection and prognostic modeling of neurological disorders. However, the clinical translation of glymphatic research remains constrained by the lack of standardized and universally validated assessment methodologies. Future researches must prioritize the establishment of reproducible imaging protocols and analytical workflows across large-scale cohorts to define unified metrics for glymphatic function. Furthermore, the integration of glymphatic indices with complementary modalities, such as electroencephalography (EEG) and CSF biomarkers, is indispensable for constructing multidimensional disease-evaluation models that enhance diagnostic precision and therapeutic monitoring.

Moreover, glymphatic assessment is positioned to serve as a cornerstone of precision therapeutics and a biomarker-informed platform for longitudinal disease trajectory tracking. For example, establishing a baseline glymphatic profile is advocated for genetically predisposed populations, such as carriers of APOE ε4 or SPPL2C variants [260, 261], and those with a significant family history. In these cohorts, diminished glymphatic efficiency relative to healthy cohorts works as a critical indicator of compromised metabolic clearance, effectively identifying individuals at heightened risk. Such early detection modalities create a pivotal window for the implementation of preventive interventions. Currently, multiple therapeutics interventions aimed at modulating the glymphatic system are under intensive exploration. BCI systems represent a frontier for personalized medicine, leveraging specific biomarkers of dysfunction to trigger precise, closed-loop neuromodulation. Non-invasive modalities, such as FUS, rTMS, 40 Hz audiovisual stimulation, have demonstrated significant potential in accelerating glymphatic circulation and are undergoing clinical validation [262–264]. Furthermore, contemporary neuroscience has transitioned from mere structural mapping to mechanistic interrogation, establishing the molecular and circuit-level evidence required for precise individual risk stratification [265]. Nevertheless, translating these experimental successes into routine clinical practice remains a formidable challenge that demands interdisciplinary collaboration across neuroscience, engineering, and clinical medicine. For example, interdisciplinary advancements in bioengineering and materials science are paramount for developing next-generation sensors with high spatiotemporal resolution to monitor glymphatic dynamics. In parallel, artificial intelligence frameworks could synergize multidimensional datasets, encompassing neuroimaging, biofluid biomarkers, genomics, and clinical metrics, to construct robust, integrative risk models. By enabling precise diagnosis and tailored therapeutics, these integrative platforms will accelerate clinical adoption of glymphatic-targeted interventions.

In conclusion, the glymphatic system serves as the primary waste clearance infrastructure of the brain, whose dysfunction constitutes a pivotal nexus linking aging, sleep disturbances, the heart-brain axis, and vascular risk factors to the onset and progression of neurological disorders. Although substantial progress has been made, the underlying pathophysiological mechanisms remain incompletely defined, and effective clinical therapeutics represent an urgent unmet need. Methodological limitations constitute the principal bottleneck. Notably, existing researches remain heavily skewed toward animal models, which exhibit anatomical and functional divergence from the human glymphatic system. In contrast, clinical investigations are largely correlational and are further hampered by single-center designs, limited sample sizes, and inconsistent findings. Large-scale, prospective clinical cohort studies with validated analytical methods are imperative. Despite its infancy, glymphatic research is poised to reshape both fundamental understanding and clinical practice in neuroscience.

## Data Availability

Not applicable. All data was obtained from published studies and it is included in the manuscript.

## References

[1] Fame RM, Lehtinen MK. Emergence and developmental roles of the cerebrospinal fluid (CSF) system. Dev Cell. 2020;52(3):261–75. 10.1016/j.devcel.2020.01.027. 32049038 10.1016/j.devcel.2020.01.027 PMC12234156

[2] Lohela TJ, Lilius TO, Nedergaard M. The glymphatic system: implications for drugs for central nervous system diseases. Nat Rev Drug Discov. 2022;21(10):763–79. 10.1038/s41573-022-00500-9. 35948785 10.1038/s41573-022-00500-9

[3] Chen J, Xiang P, Duro-Castano A, Cai H, Guo B, Liu X, et al. Rapid amyloid-β clearance and cognitive recovery through multivalent modulation of blood-brain barrier transport. Signal Transduct Target Ther. 2025;10(1):331. 10.1038/s41392-025-02426-1. 41052971 10.1038/s41392-025-02426-1 PMC12500928

[4] Franić D, Pravica M, Zubčić K, Miles S, Bedalov A, Boban M. Quiescent cells maintain active degradation-mediated protein quality control requiring proteasome, autophagy, and nucleus-vacuole junctions. J Biol Chem. 2025;301(1):108045. 10.1016/j.jbc.2024.108045. 39617269 10.1016/j.jbc.2024.108045 PMC11731230

[5] Jiang-Xie LF, Drieu A, Kipnis J. Waste clearance shapes aging brain health. Neuron. 2025;113(1):71–81. 10.1016/j.neuron.2024.09.017. 39395409 10.1016/j.neuron.2024.09.017 PMC11717645

[6] Iliff JJ, Wang M, Liao Y, Plogg BA, Peng W, Gundersen GA, et al. A paravascular pathway facilitates CSF flow through the brain parenchyma and the clearance of interstitial solutes, including amyloid β. Sci Transl Med. 2012;4(147):147ra111. 10.1126/scitranslmed.3003748. 22896675 10.1126/scitranslmed.3003748 PMC3551275

[7] Rasmussen MK, Mestre H, Nedergaard M. The glymphatic pathway in neurological disorders. Lancet Neurol. 2018;17(11):1016–24. 10.1016/S1474-4422(18)30318-1. 30353860 10.1016/S1474-4422(18)30318-1 PMC6261373

[8] Filippopulos FM, Fischer TD, Seelos K, Dunker K, Belanovic B, Crispin A, et al. Semiquantitative 3T brain magnetic resonance imaging for dynamic visualization of the glymphatic-lymphatic fluid transport system in humans: a pilot study. Invest Radiol. 2022;57(8):544–51. 10.1097/RLI.0000000000000870. 35763443 10.1097/RLI.0000000000000870

[9] Persson NDÅ, Uusalo P, Nedergaard M, Lohela TJ, Lilius TO. Could dexmedetomidine be repurposed as a glymphatic enhancer? Trends Pharmacol Sci. 2022;43(12):1030–40. 10.1016/j.tips.2022.09.007. 36280451 10.1016/j.tips.2022.09.007

[10] Da Mesquita S, Herz J, Wall M, Dykstra T, de Lima KA, Norris GT, et al. Aging-associated deficit in CCR7 is linked to worsened glymphatic function, cognition, neuroinflammation, and β-amyloid pathology. Sci Adv. 2021;7(21):eabe4601. 10.1126/sciadv.abe4601. 34020948 10.1126/sciadv.abe4601 PMC8139596

[11] Aggidis A, Devitt G, Zhang Y, Chatterjee S, Townsend D, Fullwood NJ, et al. A novel peptide-based tau aggregation inhibitor as a potential therapeutic for Alzheimer’s disease and other tauopathies. Alzheimers Dement. 2024;20(11):7788–804. 10.1002/alz.14246. 39360630 10.1002/alz.14246 PMC11567856

[12] Roemer-Cassiano SN, Wagner F, Evangelista L, Rauchmann BS, Dehsarvi A, Steward A, et al. Amyloid-associated hyperconnectivity drives tau spread across connected brain regions in Alzheimer’s disease. Sci Transl Med. 2025;17(782):eadp2564. 10.1126/scitranslmed.adp2564. 39841807 10.1126/scitranslmed.adp2564

[13] Sharkey RJ, Cortese F, Goodyear BG, Korngut LW, Jacob SM, Sharkey KA, et al. Longitudinal analysis of glymphatic function in amyotrophic lateral sclerosis and primary lateral sclerosis. Brain. 2024;147(12):4026–32. 10.1093/brain/awae288. 39241118 10.1093/brain/awae288 PMC11629681

[14] Yuan X, Nie S, Yang Y, Liu C, Xia D, Meng L, et al. Propagation of pathologic α-synuclein from kidney to brain may contribute to Parkinson’s disease. Nat Neurosci. 2025;28(3):577–88. 10.1038/s41593-024-01866-2. 39849144 10.1038/s41593-024-01866-2

[15] Ayral V, Pastor-Bernier A, Daneault V, Tremblay C, Filiatrault M, Haddad C, et al. Association of DTI-ALPS glymphatic index with differential phenoconversion in isolated REM sleep behavior disorder: a multi-cohort MRI study. Neurology. 2025;105(7):e214042. 10.1212/WNL.0000000000214042. 40956987 10.1212/WNL.0000000000214042 PMC12456434

[16] Gao M, Liu Z, Zang H, Wu X, Yan Y, Lin H, et al. A histopathologic correlation study evaluating glymphatic function in brain tumors by multiparametric MRI. Clin Cancer Res. 2024;30(21):4876–86. 10.1158/1078-0432.CCR-24-0150. 38848042 10.1158/1078-0432.CCR-24-0150 PMC11528195

[17] Huang SY, Zhang YR, Guo Y, Du J, Ren P, Wu BS, et al. Glymphatic system dysfunction predicts amyloid deposition, neurodegeneration, and clinical progression in Alzheimer’s disease. Alzheimers Dement. 2024;20(5):3251–69. 10.1002/alz.13789. 38501315 10.1002/alz.13789 PMC11095446

[18] Zhang Y, Huang G, Geng J, Li X, Xin M, Yuan P, et al. DTI-ALPS index-assessed glymphatic dysfunction mediates Alzheimer’s cognitive decline via amyloid-β-dependent pathways: multimodal PET/MRI study. Eur J Nucl Med Mol Imaging. 2025. 10.1007/s00259-025-07445-2. 40679601 10.1007/s00259-025-07445-2

[19] Feng S, Wu C, Zou P, Deng Q, Chen Z, Li M, et al. High-intensity interval training ameliorates Alzheimer’s disease-like pathology by regulating astrocyte phenotype-associated AQP4 polarization. Theranostics. 2023;13(10):3434–50. 10.7150/thno.81951. 37351177 10.7150/thno.81951 PMC10283053

[20] Li OY, Shin S, Zhou S, Turnbull A, Lin FV. Relationships between neuropsychiatric symptoms, subtypes of astrocyte activities, and brain pathologies in Alzheimer’s disease and Parkinson’s disease. Alzheimers Dement. 2025;21(5):e70242. 10.1002/alz.70242. 40390204 10.1002/alz.70242 PMC12089078

[21] Zhang Y, Zhang C, He XZ, Li ZH, Meng JC, Mao RT, et al. Interaction between the glymphatic system and α-synuclein in Parkinson’s disease. Mol Neurobiol. 2023;60(4):2209–22. 10.1007/s12035-023-03212-2. 36637746 10.1007/s12035-023-03212-2

[22] Garcia V, Blaquiere M, Janvier A, Cresto N, Lana C, Genin A, et al. PIEZO1 expression at the glio-vascular unit adjusts to neuroinflammation in seizure conditions. Neurobiol Dis. 2023;187:106297. 10.1016/j.nbd.2023.106297. 37717661 10.1016/j.nbd.2023.106297

[23] Zhang C, Xu K, Zhang H, Sha J, Yang H, Zhao H, et al. Recovery of glymphatic system function in patients with temporal lobe epilepsy after surgery. Eur Radiol. 2023;33(9):6116–23. 10.1007/s00330-023-09588-y. 37010581 10.1007/s00330-023-09588-y

[24] Guo D, Zhang B, Han L, Rensing NR, Wong M. Cerebral vascular and blood-brain-barrier abnormalities in a mouse model of epilepsy and tuberous sclerosis complex. Epilepsia. 2024;65(2):483–96. 10.1111/epi.17848. 38049961 10.1111/epi.17848 PMC10922951

[25] Feng L, Shu Y, Wu Q, Liu T, Long H, Yang H, et al. EphA4 may contribute to microvessel remodeling in the hippocampal CA1 and CA3 areas in a mouse model of temporal lobe epilepsy. Mol Med Rep. 2017;15(1):37–46. 10.3892/mmr.2016.6017. 27959424 10.3892/mmr.2016.6017 PMC5355650

[26] Andrade DM, Bassett AS, ZulfiqarAli Q, Lira VST, Reyes NG, Tartaglia MC, et al. Novel neuropathological observations in an adult with Dravet syndrome. Epilepsia. 2025;66(10):e236–41. 10.1111/epi.18613. 40956029 10.1111/epi.18613 PMC12605653

[27] Chen W, Liang C, Peng S, Bao S, Xue F, Lian X, et al. Aquaporin-4 activation facilitates glymphatic system function and hematoma clearance post-intracerebral hemorrhage. Glia. 2025;73(2):368–80. 10.1002/glia.24639. 39530196 10.1002/glia.24639 PMC11662979

[28] Gomolka RS, Hablitz LM, Mestre H, Giannetto M, Du T, Hauglund NL, et al. Loss of aquaporin-4 results in glymphatic system dysfunction via brain-wide interstitial fluid stagnation. Elife. 2023;12:e82232. 10.7554/eLife.82232. 36757363 10.7554/eLife.82232 PMC9995113

[29] Sun B, Fang D, Li W, Li M, Zhu S. NIR-II nanoprobes for investigating the glymphatic system function under anesthesia and stroke injury. J Nanobiotechnology. 2024;22(1):200. 10.1186/s12951-024-02481-w. 38654299 10.1186/s12951-024-02481-w PMC11040925

[30] Cibelli A, Ballesteros-Gomez D, McCutcheon S, Yang GL, Bispo A, et al. Astrocytes sense glymphatic-level shear stress through the interaction of sphingosine-1-phosphate with Piezo1. iScience. 2024;27(6):110069. 10.1016/j.isci.2024.110069. 38868201 10.1016/j.isci.2024.110069 PMC11167526

[31] Langen UH, Ayloo S, Gu C. Development and cell biology of the blood-brain barrier. Annu Rev Cell Dev Biol. 2019;35:591–613. 10.1146/annurev-cellbio-100617-062608. 31299172 10.1146/annurev-cellbio-100617-062608 PMC8934576

[32] Knox EG, Aburto MR, Clarke G, Cryan JF, O’Driscoll CM. The blood-brain barrier in aging and neurodegeneration. Mol Psychiatry. 2022;27(6):2659–73. 10.1038/s41380-022-01511-z. 35361905 10.1038/s41380-022-01511-z PMC9156404

[33] Mestre H, Verma N, Greene TD, Lin LA, Ladron-de-Guevara A, et al. Periarteriolar spaces modulate cerebrospinal fluid transport into brain and demonstrate altered morphology in aging and Alzheimer’s disease. Nat Commun. 2022;13(1):3897. 10.1038/s41467-022-31257-9. 35794106 10.1038/s41467-022-31257-9 PMC9259669

[34] Plog BA, Kim K, Verhaege D, Kim MW, Papadopoulos Z, et al. A route for cerebrospinal fluid flow through leptomeningeal arterial-venous overlaps enables macromolecule and fluid shunting. Nat Neurosci. 2025;28(7):1436–45. 10.1038/s41593-025-01977-4. 40490598 10.1038/s41593-025-01977-4

[35] Salman MM, Kitchen P, Halsey A, Wang MX, Törnroth-Horsefield S, et al. Emerging roles for dynamic aquaporin-4 subcellular relocalization in CNS water homeostasis. Brain. 2022;145(1):64–75. 10.1093/brain/awab311. 34499128 10.1093/brain/awab311 PMC9088512

[36] Giannetto MJ, Gomolka RS, Gahn-Martinez D, Newbold EJ, Bork PAR, et al. Glymphatic fluid transport is suppressed by the aquaporin-4 inhibitor AER-271. Glia. 2024;72(5):982–98. 10.1002/glia.24515. 38363040 10.1002/glia.24515 PMC11203403

[37] Jin H, Yoon JH, Hong SP, Hwang YS, Yang MJ, et al. Increased CSF drainage by non-invasive manipulation of cervical lymphatics. Nature. 2025;643(8072):755–67. 10.1038/s41586-025-09052-5. 40468071 10.1038/s41586-025-09052-5 PMC12267054

[38] Shen DD, Artru AA, Adkison KK. Principles and applicability of CSF sampling for the assessment of CNS drug delivery and pharmacodynamics. Adv Drug Deliv Rev. 2004;56(12):1825–57. 10.1016/j.addr.2004.07.011. 15381336 10.1016/j.addr.2004.07.011

[39] Mortensen KN, Lilius T, Rosenholm M, Sigurðsson B, Kelley DH, Nedergaard M. Perivascular cerebrospinal fluid inflow matches interstitial fluid efflux in anesthetized rats. iScience. 2025;28(5):112323. 10.1016/j.isci.2025.112323. 40271020 10.1016/j.isci.2025.112323 PMC12017870

[40] Mestre H, Tithof J, Du T, Song W, Peng W, Sweeney AM, et al. Flow of cerebrospinal fluid is driven by arterial pulsations and is reduced in hypertension. Nat Commun. 2018;9(1):4878. 10.1038/s41467-018-07318-3. 30451853 10.1038/s41467-018-07318-3 PMC6242982

[41] Xie L, Zhang Y, Hong H, Xu S, Cui L, Wang S, et al. Higher intracranial arterial pulsatility is associated with presumed imaging markers of the glymphatic system: an explorative study. Neuroimage. 2024;288:120524. 10.1016/j.neuroimage.2024.120524. 38278428 10.1016/j.neuroimage.2024.120524

[42] Nozaleda GL, Coenen W, Haughton V, Sánchez AL. Arterial pulsations and transmantle pressure synergetically drive glymphatic flow. Sci Rep. 2025;15(1):13798. 10.1038/s41598-025-97631-x. 40258946 10.1038/s41598-025-97631-x PMC12012223

[43] Rasmussen MK, Mestre H, Nedergaard M. Fluid transport in the brain. Physiol Rev. 2022;102(2):1025–51. 10.1152/physrev.00031.2020. 33949874 10.1152/physrev.00031.2020 PMC8897154

[44] Pisani F, Simone L, Mola MG, De Bellis M, Frigeri A, Nicchia GP, et al. Regulation of aquaporin-4 expression in the central nervous system investigated using M23-AQP4 null mouse. Glia. 2021;69(9):2235–51. 10.1002/glia.24032. 34038017 10.1002/glia.24032 PMC8361696

[45] de Bellis M, Cibelli A, Mola MG, Pisani F, Barile B, Mastrodonato M, et al. Orthogonal arrays of particle assembly are essential for normal aquaporin-4 expression level in the brain. Glia. 2021;69(2):473–88. 10.1002/glia.23909. 32946135 10.1002/glia.23909

[46] Pati R, Palazzo C, Valente O, Abbrescia P, Messina R, Surdo NC, et al. The readthrough isoform AQP4ex is constitutively phosphorylated in the perivascular astrocyte endfeet of human brain. Biomolecules. 2022;12(5):633. 10.3390/biom12050633. 35625560 10.3390/biom12050633 PMC9138620

[47] Zhu DD, Yang G, Huang YL, Zhang T, Sui AR, Li N, et al. AQP4-A25Q point mutation in mice depolymerizes orthogonal arrays of particles and decreases polarized expression of AQP4 protein in astrocytic endfeet at the blood-brain barrier. J Neurosci. 2022;42(43):8169–83. 10.1523/JNEUROSCI.0401-22.2022. 36100398 10.1523/JNEUROSCI.0401-22.2022 PMC9637001

[48] Yoshikawa Y, Tomioka M, Abe Y, Yasui M, Nuriya M. Early neuromyelitis optica antibody-induced molecular changes in aquaporin 4 and associated proteins at astrocyte endfeet in murine brain tissues. J Pharmacol Sci. 2025;158(3):212–8. 10.1016/j.jphs.2025.05.007. 40436483 10.1016/j.jphs.2025.05.007

[49] Kato D, Kameda H, Kinota N, Fujii T, Xiawei B, Simi Z, et al. Loss of aquaporin-4 impairs cerebrospinal fluid solute clearance through cerebrospinal fluid drainage pathways. Sci Rep. 2024;14(1):27982. 10.1038/s41598-024-79147-y. 39543281 10.1038/s41598-024-79147-y PMC11564557

[50] Zhang H, Wang J, Zhang S, Yan D, Dong Y, Zhang P, et al. Aquaporin 4 and its isoforms regulation ameliorate AQP4 mis-localization-induced glymphatic dysfunction in ischemic stroke. J Adv Res. 2025 [Epub ahead of print]. 10.1016/j.jare.2025.05.022. 10.1016/j.jare.2025.05.022 PMC12869294 40403843

[51] Huang H, Lin L, Wu T, Wu C, Zhou L, Li G, et al. Phosphorylation of AQP4 by LRRK2 R1441G impairs glymphatic clearance of IFNγ and aggravates dopaminergic neurodegeneration. NPJ Park Dis. 2024;10(1):31. 10.1038/s41531-024-00643-z. 10.1038/s41531-024-00643-z PMC10831045 38296953

[52] Jeon H, Kim M, Park W, Lim JS, Lee E, Cha H, et al. Upregulation of AQP4 improves blood-brain barrier integrity and perihematomal edema following intracerebral hemorrhage. Neurotherapeutics. 2021;18(4):2692–706. 10.1007/s13311-021-01126-2. 34545550 10.1007/s13311-021-01126-2 PMC8804112

[53] Geng Z, Deng G, Wang Z, Xu X, Yin X, Zhang S, et al. Distinct roles of A1/A2 astrocytes in blood-brain barrier injury following cerebral I/R via the ROCK/NF-κB and STAT3 pathways. Int Immunopharmacol. 2025;151:114338. 10.1016/j.intimp.2025.114338. 40020465 10.1016/j.intimp.2025.114338

[54] Zhao Z, He J, Chen Y, Wang Y, Wang C, Tan C, et al. The pathogenesis of idiopathic normal pressure hydrocephalus based on the understanding of AQP1 and AQP4. Front Mol Neurosci. 2022;15:952036. 10.3389/fnmol.2022.952036. 36204139 10.3389/fnmol.2022.952036 PMC9530743

[55] Wang H, Zheng X, Jin J, Zheng L, Guan T, Huo Y, et al. LncRNA MALAT1 silencing protects against cerebral ischemia-reperfusion injury through miR-145 to regulate AQP4. J Biomed Sci. 2020;27(1):40. 10.1186/s12929-020-00635-0. 32138732 10.1186/s12929-020-00635-0 PMC7059719

[56] Zhang J, Tian Y, Wang R, Qin X, Li Q, Ma K, et al. Sevoflurane reduces brain edema and improves blood-brain barrier by downregulating CaMKII to inhibit TMEM16A after cerebral ischemia injury both in vivo and in vitro. Brain Res Bull. 2025;229:111479. 10.1016/j.brainresbull.2025.111479. 40714175 10.1016/j.brainresbull.2025.111479

[57] Chen C, Zhu B, Luo W, Cao A, Zhou W, Weng Y, et al. Trifluoperazine improves postoperative cognition by influencing astrocyte endfoot morphology and aquaporin-4 polarity. Mol Neurobiol. 2025;62(10):12574–87. 10.1007/s12035-025-05072-4. 40425909 10.1007/s12035-025-05072-4

[58] Sun H, Cao Q, He X, Du X, Jiang X, Wu T, et al. Melatonin mitigates sleep restriction-induced cognitive and glymphatic dysfunction via aquaporin-4 polarization. Mol Neurobiol. 2025;62(9):11443–65. 10.1007/s12035-025-04992-5. 40293704 10.1007/s12035-025-04992-5

[59] Lopes DM, Wells JA, Ma D, Wallis L, Park D, Llewellyn SK, et al. Glymphatic inhibition exacerbates tau propagation in an Alzheimer’s disease model. Alzheimers Res Ther. 2024;16(1):71. 10.1186/s13195-024-01439-2. 38576025 10.1186/s13195-024-01439-2 PMC10996277

[60] Lu H, Ai L, Zhang B. TNF-α induces AQP4 overexpression in astrocytes through the NF-κB pathway causing cellular edema and apoptosis. Biosci Rep. 2022;42(3):BSR20212224. 10.1042/BSR20212224. 35260880 10.1042/BSR20212224 PMC8935387

[61] Wang S, Yu X, Cheng L, Ren W, Wen G, Wu X, et al. Dexmedetomidine improves the circulatory dysfunction of the glymphatic system induced by sevoflurane through the PI3K/AKT/ΔFosB/AQP4 pathway in young mice. Cell Death Dis. 2024;15(6):448. 10.1038/s41419-024-06845-w. 38918408 10.1038/s41419-024-06845-w PMC11199640

[62] Wu CH, Liao WH, Chu YC, Hsiao MY, Kung Y, Wang JL, et al. Very low-intensity ultrasound facilitates glymphatic influx and clearance via modulation of the TRPV4-AQP4 pathway. Adv Sci (Weinh). 2024;11(47):e2401039. 10.1002/advs.202401039. 39494466 10.1002/advs.202401039 PMC11653672

[63] Sun X, Dias L, Peng C, Zhang Z, Ge H, Wang Z, et al. 40 Hz light flickering facilitates the glymphatic flow via adenosine signaling in mice. Cell Discov. 2024;10(1):81. 10.1038/s41421-024-00701-z. 39103336 10.1038/s41421-024-00701-z PMC11300858

[64] Siow TY, Toh CH, Hsu JL, Liu GH, Lee SH, Chen NH, et al. Association of sleep, neuropsychological performance, and gray matter volume with glymphatic function in community-dwelling older adults. Neurology. 2022;98(8):e829–38. 10.1212/WNL.0000000000013215. 34906982 10.1212/WNL.0000000000013215

[65] Li W, Chen D, Liu N, Luan Y, Zhu S, Wang H. Modulation of lymphatic transport in the central nervous system. Theranostics. 2022;12(3):1117–31. 10.7150/thno.66026. 35154477 10.7150/thno.66026 PMC8771567

[66] Beschorner N, Nedergaard M. Glymphatic system dysfunction in neurodegenerative diseases. Curr Opin Neurol. 2024;37(2):182–8. 10.1097/WCO.0000000000001252. 38345416 10.1097/WCO.0000000000001252

[67] Hauglund NL, Andersen M, Tokarska K, Radovanovic T, Kjaerby C, Sørensen FL, et al. Norepinephrine-mediated slow vasomotion drives glymphatic clearance during sleep. Cell. 2025;188(3):606-22.e17. 10.1016/j.cell.2024.11.027. 39788123 10.1016/j.cell.2024.11.027 PMC12340670

[68] Ma J, Chen M, Liu GH, Gao M, Chen NH, Toh CH, et al. Effects of sleep on the glymphatic functioning and multimodal human brain network affecting memory in older adults. Mol Psychiatry. 2025;30(5):1717–29. 10.1038/s41380-024-02778-0. 39397082 10.1038/s41380-024-02778-0 PMC12014484

[69] Cankar N, Beschorner N, Tsopanidou A, Qvist FL, Colaço AR, Andersen M, et al. Sleep deprivation leads to non-adaptive alterations in sleep microarchitecture and amyloid-β accumulation in a murine Alzheimer model. Cell Rep. 2024;43(11):114977. 10.1016/j.celrep.2024.114977. 39541211 10.1016/j.celrep.2024.114977 PMC12379227

[70] Shokri-Kojori E, Wang GJ, Wiers CE, Demiral SB, Guo M, Kim SW, et al. β-Amyloid accumulation in the human brain after one night of sleep deprivation. Proc Natl Acad Sci U S A. 2018;115(17):4483–8. 10.1073/pnas.1721694115. 29632177 10.1073/pnas.1721694115 PMC5924922

[71] Kjaerby C, Andersen M, Hauglund N, Untiet V, Dall C, Sigurdsson B, et al. Memory-enhancing properties of sleep depend on the oscillatory amplitude of norepinephrine. Nat Neurosci. 2022;25(8):1059–70. 10.1038/s41593-022-01102-9. 35798980 10.1038/s41593-022-01102-9 PMC9817483

[72] Koshmanova E, Berger A, Beckers E, Campbell I, Mortazavi N, Sharifpour R, et al. Locus coeruleus activity while awake is associated with REM sleep quality in older individuals. JCI Insight. 2023;8(20):e172008. 10.1172/jci.insight.172008. 37698926 10.1172/jci.insight.172008 PMC10619502

[73] Xie Z, He Z, Yuan Z, Wang M, Zhou F. The regulation of cerebral lymphatic drainage in the transverse sinus region of the mouse brain. J Biophotonics. 2024. 10.1002/jbio.202400250. 39289863 10.1002/jbio.202400250

[74] Lüthi A, Nedergaard M. Anything but small: microarousals stand at the crossroad between noradrenaline signaling and key sleep functions. Neuron. 2025;113(4):509–23. 10.1016/j.neuron.2024.12.009. 39809276 10.1016/j.neuron.2024.12.009

[75] Örzsik B, Palombo M, Asllani I, Dijk DJ, Harrison NA, Cercignani M. Higher order diffusion imaging as a putative index of human sleep-related microstructural changes and glymphatic clearance. Neuroimage. 2023;274:120124. 10.1016/j.neuroimage.2023.120124. 37084927 10.1016/j.neuroimage.2023.120124

[76] Morawska MM, Moreira CG, Ginde VR, Valko PO, Weiss T, Büchele F, et al. Slow-wave sleep affects synucleinopathy and regulates proteostatic processes in mouse models of Parkinson’s disease. Sci Transl Med. 2021;13(623):eabe7099. 10.1126/scitranslmed.abe7099. 34878820 10.1126/scitranslmed.abe7099

[77] Himali JJ, Baril AA, Cavuoto MG, Yiallourou S, Wiedner CD, Himali D, et al. Association between slow-wave sleep loss and incident dementia. JAMA Neurol. 2023;80(12):1326–33. 10.1001/jamaneurol.2023.3889. 37902739 10.1001/jamaneurol.2023.3889 PMC10616771

[78] Hablitz LM, Plá V, Giannetto M, Vinitsky HS, Stæger FF, Metcalfe T, et al. Circadian control of brain glymphatic and lymphatic fluid flow. Nat Commun. 2020;11(1):4411. 10.1038/s41467-020-18115-2. 32879313 10.1038/s41467-020-18115-2 PMC7468152

[79] Vasciaveo V, Iadarola A, Casile A, Dante D, Morello G, Minotta L, et al. Sleep fragmentation affects glymphatic system through the different expression of AQP4 in wild type and 5xFAD mouse models. Acta Neuropathol Commun. 2023;11(1):16. 10.1186/s40478-022-01498-2. 36653878 10.1186/s40478-022-01498-2 PMC9850555

[80] Cruz-Sanabria F, Bruno S, Crippa A, Frumento P, Scarselli M, Skene DJ, et al. Optimizing the time and dose of melatonin as a sleep-promoting drug: a systematic review of randomized controlled trials and dose-response meta-analysis. J Pineal Res. 2024;76(5):e12985. 10.1111/jpi.12985. 38888087 10.1111/jpi.12985

[81] Chen Y, Guo H, Sun X, Wang S, Zhao M, Gong J, et al. Melatonin regulates glymphatic function to affect cognitive deficits, behavioral issues, and blood-brain barrier damage in mice after intracerebral hemorrhage: potential links to circadian rhythms. CNS Neurosci Ther. 2025;31(2):e70289. 10.1111/cns.70289. 39981743 10.1111/cns.70289 PMC11843476

[82] Yao D, Li R, Hao J, Huang H, Wang X, Ran L, et al. Melatonin alleviates depression-like behaviors and cognitive dysfunction in mice by regulating the circadian rhythm of AQP4 polarization. Transl Psychiatry. 2023;13(1):310. 10.1038/s41398-023-02614-z. 37802998 10.1038/s41398-023-02614-z PMC10558463

[83] Han G, Jiao B, Zhang Y, Wang Z, Liang C, Li Y, et al. Arterial pulsation dependence of perivascular cerebrospinal fluid flow measured by dynamic diffusion tensor imaging in the human brain. Neuroimage. 2024;297:120653. 10.1016/j.neuroimage.2024.120653. 38795798 10.1016/j.neuroimage.2024.120653

[84] Sharp MK. Pulsatile cerebral paraarterial flow by peristalsis, pressure and directional resistance. Fluids Barriers CNS. 2023;20(1):41. 10.1186/s12987-023-00445-0. 37291600 10.1186/s12987-023-00445-0 PMC10249285

[85] Buongiorno M, Sánchez-Benavides G, Caruana G, Elias-Mas A, Artero C, et al. Abnormal sleep blood pressure patterns are associated with the diffusion tensor imaging along the perivascular space index in cognitively impaired individuals. Front Aging Neurosci. 2025;17:1578270. 10.3389/fnagi.2025.1578270. 40951923 10.3389/fnagi.2025.1578270 PMC12426274

[86] Hofer S, Schnieder M, Polster L, Dechent P, Bähr M. Atrial fibrillation reduces CSF flow dynamics: a multimodal MRI study. Neuroimage. 2025;317:121337. 10.1016/j.neuroimage.2025.121337. 40554030 10.1016/j.neuroimage.2025.121337

[87] Zheng C, Cui Y, Qin R, Si J, Xiao K, Li G, et al. Association of glymphatic system dysfunction with cardiac injury and cognitive impairment in heart failure: a multimodal MRI study. Acad Radiol. 2025;32(6):3227–36. 10.1016/j.acra.2025.03.043. 40210519 10.1016/j.acra.2025.03.043

[88] Guo J, Zhang Z, Meng X, Jing J, Hu Y, Yao Y, et al. Atrial fibrillation catheter ablation, brain glymphatic function, and cognitive performance. Eur Heart J. 2025;46(18):1733–43. 10.1093/eurheartj/ehaf036. 39981927 10.1093/eurheartj/ehaf036

[89] Bernier LP, Hefendehl JK, Scott RW, Tung LW, Lewis CA, Soliman H, et al. Brain pericytes and perivascular fibroblasts are stromal progenitors with dual functions in cerebrovascular regeneration after stroke. Nat Neurosci. 2025;28(3):517–35. 10.1038/s41593-025-01872-y. 39962273 10.1038/s41593-025-01872-y

[90] Yang AC, Vest RT, Kern F, Lee DP, Agam M, Maat CA, et al. A human brain vascular atlas reveals diverse mediators of Alzheimer’s risk. Nature. 2022;603(7903):885–92. 10.1038/s41586-021-04369-3. 35165441 10.1038/s41586-021-04369-3 PMC9635042

[91] Goodman JR, Iliff JJ. Vasomotor influences on glymphatic-lymphatic coupling and solute trafficking in the central nervous system. J Cereb Blood Flow Metab. 2020;40(8):1724–34. 10.1177/0271678X19874134. 31506012 10.1177/0271678X19874134 PMC7370362

[92] Dreha-Kulaczewski S, Joseph AA, Merboldt KD, Ludwig HC, Gärtner J, Frahm J. Identification of the upward movement of human CSF in vivo and its relation to the brain venous system. J Neurosci. 2017;37(9):2395–402. 10.1523/JNEUROSCI.2754-16.2017. 28137972 10.1523/JNEUROSCI.2754-16.2017 PMC6596847

[93] Vinje V, Ringstad G, Lindstrøm EK, Valnes LM, Rognes ME, Eide PK, et al. Respiratory influence on cerebrospinal fluid flow-a computational study based on long-term intracranial pressure measurements. Sci Rep. 2019;9(1):9732. 10.1038/s41598-019-46055-5. 31278278 10.1038/s41598-019-46055-5 PMC6611841

[94] Ozturk B, Koundal S, Al Bizri E, Chen X, Gursky Z, Dai F, et al. Continuous positive airway pressure increases CSF flow and glymphatic transport. JCI Insight. 2023;8(12):e170270. 10.1172/jci.insight.170270. 37159262 10.1172/jci.insight.170270 PMC10371231

[95] Romay MC, Knutsen RH, Ma F, Mompeón A, Hernandez GE, et al. Age-related loss of Notch3 underlies brain vascular contractility deficiencies, glymphatic dysfunction, and neurodegeneration in mice. J Clin Invest. 2024;134(2):e166134. 10.1172/JCI166134. 38015629 10.1172/JCI166134 PMC10786701

[96] Yoon JH, Jin H, Kim HJ, Hong SP, Yang MJ, Ahn JH, et al. Nasopharyngeal lymphatic plexus is a hub for cerebrospinal fluid drainage. Nature. 2024;625(7996):768–77. 10.1038/s41586-023-06899-4. 38200313 10.1038/s41586-023-06899-4 PMC10808075

[97] Jin H, Yoon JH, Hong SP, Hwang YS, Yang MJ, Choi J, et al. Increased CSF drainage by non-invasive manipulation of cervical lymphatics. Nature. 2025;643(8072):755–67. 10.1038/s41586-025-09052-5. 40468071 10.1038/s41586-025-09052-5 PMC12267054

[98] Labarta-Bajo L, Allen NJ. Astrocytes in aging. Neuron. 2025;113(1):109–26. 10.1016/j.neuron.2024.12.010. 39788083 10.1016/j.neuron.2024.12.010 PMC11735045

[99] Zhang Z, Ren H, Cheng Y, Qiu H, Luo Q, Zhao Y, et al. Sleep disorders and aging: Mendelian randomization analysis of epigenetic and frailty markers. J Affect Disord. 2025;391:119989. 10.1016/j.jad.2025.119989. 40738335 10.1016/j.jad.2025.119989

[100] Zaheed AB, Tapia AL, Oryshkewych N, Wheeler BJ, Butters MA, Buysse DJ, et al. Sleep trajectories across three cognitive-aging pathways in community older adults. Alzheimers Dement. 2025;21(5):e70159. 10.1002/alz.70159. 40317639 10.1002/alz.70159 PMC12046567

[101] Zhang D, Ruan J, Peng S, Li J, Hu X, Zhang Y, et al. Synaptic-like transmission between neural axons and arteriolar smooth muscle cells drives cerebral neurovascular coupling. Nat Neurosci. 2024;27(2):232–48. 10.1038/s41593-023-01515-0. 38168932 10.1038/s41593-023-01515-0 PMC10849963

[102] Yao D, Zhang R, Xie M, Ding F, Wang M, Wang W. Updated understanding of the glial-vascular unit in central nervous system disorders. Neurosci Bull. 2023;39(3):503–18. 10.1007/s12264-022-00977-9. 36374471 10.1007/s12264-022-00977-9 PMC10043098

[103] Gundersen GA, Vindedal GF, Skare O, Nagelhus EA. Evidence that pericytes regulate aquaporin-4 polarization in mouse cortical astrocytes. Brain Struct Funct. 2014;219(6):2181–6. 10.1007/s00429-013-0629-0. 23982198 10.1007/s00429-013-0629-0 PMC4223569

[104] Fultz NE, Bonmassar G, Setsompop K, Stickgold RA, Rosen BR, Polimeni JR, et al. Coupled electrophysiological, hemodynamic, and cerebrospinal fluid oscillations in human sleep. Science. 2019;366(6465):628–31. 10.1126/science.aax5440. 31672896 10.1126/science.aax5440 PMC7309589

[105] Turner KL, Gheres KW, Proctor EA, Drew PJ. Neurovascular coupling and bilateral connectivity during NREM and REM sleep. Elife. 2020;9:e62071. 10.7554/eLife.62071. 33118932 10.7554/eLife.62071 PMC7758068

[106] Holstein-Rønsbo S, Gan Y, Giannetto MJ, Rasmussen MK, Sigurdsson B, Beinlich FRM, et al. Glymphatic influx and clearance are accelerated by neurovascular coupling. Nat Neurosci. 2023;26(6):1042–53. 10.1038/s41593-023-01327-2. 37264158 10.1038/s41593-023-01327-2 PMC10500159

[107] Kumar AA, Yeo N, Whittaker M, Attra P, Barrick TR, Bridges LR, et al. Vascular collagen type-IV in hypertension and cerebral small vessel disease. Stroke. 2022;53(12):3696–705. 10.1161/STROKEAHA.122.037761. 36205142 10.1161/STROKEAHA.122.037761 PMC9698121

[108] Zhao Y, Zhang P, Zhang J. Microglia-mediated endothelial protection: the role of SHPL-49 in ischemic stroke. Biomed Pharmacother. 2024;180:117530. 10.1016/j.biopha.2024.117530. 39388998 10.1016/j.biopha.2024.117530

[109] Hartmann DA, Berthiaume AA, Grant RI, Harrill SA, Koski T, Tieu T, et al. Brain capillary pericytes exert a substantial but slow influence on blood flow. Nat Neurosci. 2021;24(5):633–45. 10.1038/s41593-020-00793-2. 33603231 10.1038/s41593-020-00793-2 PMC8102366

[110] Yang Y, Zhang Q, Liu S, Yuan H, Wu X, Zou Y, et al. Suv39h1 regulates phenotypic modulation of smooth muscle cells and contributes to vascular injury by repressing HIC1 transcription. Arterioscler Thromb Vasc Biol. 2025;45(6):965–78. 10.1161/ATVBAHA.124.322048. 40308197 10.1161/ATVBAHA.124.322048 PMC12094260

[111] Khaddaj-Mallat R, Aldib N, Bernard M, Paquette AS, Ferreira A, Lecordier S, et al. SARS-CoV-2 deregulates the vascular and immune functions of brain pericytes via Spike protein. Neurobiol Dis. 2021;161:105561. 10.1016/j.nbd.2021.105561. 34780863 10.1016/j.nbd.2021.105561 PMC8590447

[112] Lin L, Tang H, Cui K, Kang Z, Pan T, Feng C, et al. The PDGFBB-PDGFRβ pathway and laminins in pericytes are involved in the temporal change of AQP4 polarity during temporal lobe epilepsy pathogenesis. eNeuro. 2025;12(10):ENEURO.0196-25.2025. 10.1523/ENEURO.0196-25.2025. 41057266 10.1523/ENEURO.0196-25.2025 PMC12669438

[113] Lin L, Hu X, Hong W, Pan T, Wang Z, Wang E, et al. A novel animal model of spontaneous epilepsy: Cdk5 knockout in pericyte-specific mice. Front Cell Neurosci. 2024;18:1474231. 10.3389/fncel.2024.1474231. 39479522 10.3389/fncel.2024.1474231 PMC11521856

[114] Korte N, Barkaway A, Wells J, Freitas F, Sethi H, Andrews SP, et al. Inhibiting Ca2⁺ channels in Alzheimer’s disease model mice relaxes pericytes, improves cerebral blood flow and reduces immune cell stalling and hypoxia. Nat Neurosci. 2024;27(11):2086–100. 10.1038/s41593-024-01753-w. 39294491 10.1038/s41593-024-01753-w PMC11537984

[115] Kyriatzis G, Bernard A, Bôle A, Khrestchatisky M, Ferhat L. In the rat hippocampus, pilocarpine-induced status epilepticus is associated with reactive glia and concomitant increased expression of CD31, PDGFRβ, and collagen IV in endothelial cells and pericytes of the blood-brain barrier. Int J Mol Sci. 2024;25(3):1693. 10.3390/ijms25031693. 38338969 10.3390/ijms25031693 PMC10855308

[116] Tsao CC, Baumann J, Huang SF, Kindler D, Schroeter A, Kachappilly N, et al. Pericyte hypoxia-inducible factor-1 (HIF-1) drives blood-brain barrier disruption and impacts acute ischemic stroke outcome. Angiogenesis. 2021;24(4):823–42. 10.1007/s10456-021-09796-4. 34046769 10.1007/s10456-021-09796-4 PMC8487886

[117] Wan H, Brathwaite S, Ai J, Hynynen K, Macdonald RL. Role of perivascular and meningeal macrophages in outcome following experimental subarachnoid hemorrhage. J Cereb Blood Flow Metab. 2021;41(8):1842–57. 10.1177/0271678X20980296. 33444089 10.1177/0271678X20980296 PMC8327101

[118] Li C, Li T, Hu M, Kang X, Su X, Wang S, et al. Novel perivascular macrophage mechanism to promote glymphatic Aβ clearance after stroke. Stroke. 2025;56(9):2695–706. 10.1161/STROKEAHA.124.050266. 40391473 10.1161/STROKEAHA.124.050266

[119] Lau V, VanderZwaag J, Murray CJ, Tremblay MÈ. Exploring iron deposition patterns using light and electron microscopy in the mouse brain across aging and Alzheimer’s disease pathology conditions. J Neurochem. 2025;169(6):e70086. 10.1111/jnc.70086. 40469030 10.1111/jnc.70086 PMC12138743

[120] Silvin A, Qian J, Ginhoux F. Brain macrophage development, diversity and dysregulation in health and disease. Cell Mol Immunol. 2023;20(11):1277–89. 10.1038/s41423-023-01053-6. 37365324 10.1038/s41423-023-01053-6 PMC10616292

[121] Carder JD, Barile B, Memeo A, Jacobo EP, Nicchia GP, Brozik JA. Size of aquaporin-4 orthogonal arrays of particles are affected by the palmitoylation state of the M1 isoform. Biochim Biophys Acta Gen Subj. 2025;1869(12):130866. 10.1016/j.bbagen.2025.130866. 41077234 10.1016/j.bbagen.2025.130866

[122] Dias L, Nabais AM, Borges-Martins VPP, Canas PM, Cunha RA, Agostinho P. Impact of glucocorticoid-associated stress-like conditions on aquaporin-4 in cultured astrocytes and its modulation by adenosine A2A receptors. J Neurochem. 2025;169(1):e16299. 10.1111/jnc.16299. 39754374 10.1111/jnc.16299

[123] Belmaati Cherkaoui M, Vacca O, Izabelle C, Boulay AC, Boulogne C, et al. Dp71 contribution to the molecular scaffold anchoring aquaporine‐4 channels in brain macroglial cells. Glia. 2021;69(4):954–70. 10.1002/glia.23941. 33247858 10.1002/glia.23941

[124] Wang C, Li L. The critical role of KLF4 in regulating the activation of A1/A2 reactive astrocytes following ischemic stroke. J Neuroinflammation. 2023;20(1):44. 10.1186/s12974-023-02742-9. 36823628 10.1186/s12974-023-02742-9 PMC9948409

[125] Feng XF, Li MC, Lin ZY, Li MZ, Lu Y, Zhuang YM, et al. Tetramethylpyrazine promotes stroke recovery by inducing the restoration of neurovascular unit and transformation of A1/A2 reactive astrocytes. Front Cell Neurosci. 2023;17:1125412. 10.3389/fncel.2023.1125412. 37051111 10.3389/fncel.2023.1125412 PMC10083399

[126] Si X, Dai S, Fang Y, Tang J, Wang Z, Li Y, et al. Matrix metalloproteinase-9 inhibition prevents aquaporin-4 depolarization-mediated glymphatic dysfunction in Parkinson’s disease. J Adv Res. 2024;56:125–36. 10.1016/j.jare.2023.03.004. 36940850 10.1016/j.jare.2023.03.004 PMC10834796

[127] Moëlo C, Quillévéré A, Le Roy L, Timsit S. (S)-roscovitine, a CDK inhibitor, decreases cerebral edema and modulates AQP4 and α1-syntrophin interaction on a pre-clinical model of acute ischemic stroke. Glia. 2024;72(2):322–37. 10.1002/glia.24477. 37828900 10.1002/glia.24477

[128] Fei X, Dou YN, Wang L, Wu X, Huan Y, Wu S, et al. Homer1 promotes the conversion of A1 astrocytes to A2 astrocytes and improves the recovery of transgenic mice after intracerebral hemorrhage. J Neuroinflammation. 2022;19(1):67. 10.1186/s12974-022-02428-8. 35287697 10.1186/s12974-022-02428-8 PMC8922810

[129] Landmesser U, Skurk C, Tzikas A, Falk V, Reddy VY, Windecker S. Left atrial appendage closure for stroke prevention in atrial fibrillation: current status and perspectives. Eur Heart J. 2024;45(32):2914–32. 10.1093/eurheartj/ehae398. 39027946 10.1093/eurheartj/ehae398 PMC11335376

[130] Wang J, Huang P, Yu Q, Lu J, Liu P, Yang Y, et al. Epilepsy and long-term risk of arrhythmias. Eur Heart J. 2023;44(35):3374–82. 10.1093/eurheartj/ehad523. 37602368 10.1093/eurheartj/ehad523 PMC10499547

[131] Sarmiento JVM, Casis RM, Opinaldo PVA. Understanding the brain-heart connection through a case of angry glioma syndrome. Brain Tumor Res Treat. 2024;12(2):121–4. 10.14791/btrt.2024.0004. 38742261 10.14791/btrt.2024.0004 PMC11096629

[132] Kritsilis M, Vanherle L, Rosenholm M, In ’t Zandt R, Yao Y, Swanberg KM, et al. Loss of glymphatic homeostasis in heart failure. Brain. 2025;148(3):985–1000. 10.1093/brain/awae411. 39693238 10.1093/brain/awae411 PMC11884761

[133] Valenza G, Matić Z, Catrambone V. The brain-heart axis: integrative cooperation of neural, mechanical and biochemical pathways. Nat Rev Cardiol. 2025;22(8):537–50. 10.1038/s41569-025-01140-3. 40033035 10.1038/s41569-025-01140-3

[134] Shi ZM, Jing JJ, Xue ZJ, Chen WJ, Tang YB, Chen DJ, et al. Stellate ganglion block ameliorated central post-stroke pain with comorbid anxiety and depression through inhibiting HIF-1α/NLRP3 signaling following thalamic hemorrhagic stroke. J Neuroinflammation. 2023;20(1):82. 10.1186/s12974-023-02765-2. 36944982 10.1186/s12974-023-02765-2 PMC10031944

[135] Wittenberg P, McBryde FD, Korsak A, Rodrigues KL, Paton JFR, Marina N, et al. On the regulation of arterial blood pressure by an intracranial baroreceptor mechanism. J Physiol. 2025;603(9):2517–32. 10.1113/JP285082. 39924875 10.1113/JP285082 PMC7617620

[136] Jammal Salameh L, Bitzenhofer SH, Hanganu-Opatz IL, Dutschmann M, Egger V. Blood pressure pulsations modulate central neuronal activity via mechanosensitive ion channels. Science. 2024;383(6682):eadk8511. 10.1126/science.adk8511. 38301001 10.1126/science.adk8511

[137] Choi D, Park E, Yu RP, Cooper MN, Cho IT, Choi J, et al. Piezo1-regulated mechanotransduction controls flow-activated lymphatic expansion. Circ Res. 2022;131(2):e2–21. 10.1161/CIRCRESAHA.121.320565. 35701867 10.1161/CIRCRESAHA.121.320565 PMC9308715

[138] Matrongolo MJ, Ang PS, Wu J, Jain A, Thackray JK, Reddy A, et al. Piezo1 agonist restores meningeal lymphatic vessels, drainage, and brain-CSF perfusion in craniosynostosis and aged mice. J Clin Invest. 2023;134(4):e171468. 10.1172/JCI171468. 37917195 10.1172/JCI171468 PMC10866656

[139] Yuksel D, Kiss O, Prouty D, Arra N, Volpe L, Baker FC, et al. Stress, hypothalamic pituitary adrenal axis activity and autonomic nervous system function in adolescents with insomnia. Int J Psychophysiol. 2023;187:43–53. 10.1016/j.ijpsycho.2023.02.006. 36822502 10.1016/j.ijpsycho.2023.02.006 PMC10041935

[140] Salardini A, Himali JJ, Abdullah MS, Chaudhari R, Young V, Zilli EM, et al. Elevated serum cortisol associated with early-detected increase of brain amyloid deposition in Alzheimer’s disease imaging biomarkers among menopausal women: the Framingham Heart Study. Alzheimers Dement. 2025;21(4):e70179. 10.1002/alz.70179. 40271551 10.1002/alz.70179 PMC12019305

[141] Montellano FA, Ungethüm K, Ramiro L, Nacu A, Hellwig S, Fluri F, et al. Role of blood-based biomarkers in ischemic stroke prognosis: a systematic review. Stroke. 2021;52(2):543–51. 10.1161/STROKEAHA.120.029232. 33430636 10.1161/STROKEAHA.120.029232

[142] Mohd Azmi NAS, Juliana N, Azmani S, Mohd Effendy N, Abu IF, Mohd Fahmi Teng NI, et al. Cortisol on circadian rhythm and its effect on cardiovascular system. Int J Environ Res Public Health. 2021;18(2):676. 10.3390/ijerph18020676. 33466883 10.3390/ijerph18020676 PMC7830980

[143] Lin HB, Hong P, Yin MY, Yao ZJ, Zhang JY, Jiang YP, et al. Monocyte-derived macrophages aggravate cardiac dysfunction after ischemic stroke in mice. J Am Heart Assoc. 2024;13(9):e034731. 10.1161/JAHA.123.034731. 38700011 10.1161/JAHA.123.034731 PMC11179859

[144] Fischer S, Nasyrov E, Brosien M, Preissner KT, Marti HH, Kunze R. Self-extracellular RNA promotes pro-inflammatory response of astrocytes to exogenous and endogenous danger signals. J Neuroinflammation. 2021;18(1):252. 10.1186/s12974-021-02286-w. 34727934 10.1186/s12974-021-02286-w PMC8561902

[145] Barca C, Wiesmann M, Calahorra J, Wachsmuth L, Döring C, et al. Impact of hydroxytyrosol on stroke: tracking therapy response on neuroinflammation and cerebrovascular parameters using PET-MR imaging and on functional outcomes. Theranostics. 2021;11(9):4030–49. 10.7150/thno.48110. 33754046 10.7150/thno.48110 PMC7977466

[146] Wu L, Liu Y, He Q, Ao G, Xu N, He W, et al. PEDF-34 attenuates neurological deficit and suppresses astrocyte-dependent neuroinflammation by modulating astrocyte polarization via 67LR/JNK/STAT1 signaling pathway after subarachnoid hemorrhage in rats. J Neuroinflammation. 2024;21(1):178. 10.1186/s12974-024-03171-y. 39034417 10.1186/s12974-024-03171-y PMC11264993

[147] Liddelow SA, Guttenplan KA, Clarke LE, Bennett FC, Bohlen CJ, Schirmer L, et al. Neurotoxic reactive astrocytes are induced by activated microglia. Nature. 2017;541(7638):481–7. 10.1038/nature21029. 28099414 10.1038/nature21029 PMC5404890

[148] Khandelwal M, Ying Z, Gomez-Pinilla F. Thyroid hormone T4 alleviates traumatic brain injury by enhancing blood-brain barrier integrity. Int J Mol Sci. 2025;26(19):9632. 10.3390/ijms26199632. 41096912 10.3390/ijms26199632 PMC12524614

[149] Wang J, Wu Y, Chen J, Zhang Q, Liu Y, Long H, et al. Th1/Th2 imbalance in peripheral blood echoes microglia state dynamics in CNS during TLE progression. Adv Sci (Weinh). 2024;11(39):e2405346. 10.1002/advs.202405346. 39136073 10.1002/advs.202405346 PMC11496985

[150] Ma Y, Zheng K, Zhao C, Chen J, Chen L, Zhang Y, et al. Microglia LILRB4 upregulation reduces brain damage after acute ischemic stroke by limiting CD8+ T cell recruitment. J Neuroinflammation. 2024;21(1):214. 10.1186/s12974-024-03206-4. 39217343 10.1186/s12974-024-03206-4 PMC11366150

[151] Zeng Q, Li K, Luo X, Wang S, Xu X, Jiaerken Y, et al. The association of enlarged perivascular space with microglia-related inflammation and Alzheimer’s pathology in cognitively normal elderly. Neurobiol Dis. 2022;170:105755. 10.1016/j.nbd.2022.105755. 35577066 10.1016/j.nbd.2022.105755

[152] He XF, Li LL, Xian WB, Li MY, Zhang LY, Xu JH, et al. Chronic colitis exacerbates NLRP3-dependent neuroinflammation and cognitive impairment in middle-aged brain. J Neuroinflammation. 2021;18(1):153. 10.1186/s12974-021-02199-8. 34229722 10.1186/s12974-021-02199-8 PMC8262017

[153] Mai CL, Tan Z, Xu YN, Zhang JJ, Huang ZH, Wang D, et al. CXCL12-mediated monocyte transmigration into brain perivascular space leads to neuroinflammation and memory deficit in neuropathic pain. Theranostics. 2021;11(3):1059–78. 10.7150/thno.44364. 33391521 10.7150/thno.44364 PMC7738876

[154] Bolte AC, Dutta AB, Hurt ME, Smirnov I, Kovacs MA, McKee CA, et al. Meningeal lymphatic dysfunction exacerbates traumatic brain injury pathogenesis. Nat Commun. 2020;11:4524. 10.1038/s41467-020-18113-4. 32913280 10.1038/s41467-020-18113-4 PMC7483525

[155] Simon M, Wang MX, Ismail O, Braun M, Schindler AG, Reemmer J, et al. Loss of perivascular aquaporin-4 localization impairs glymphatic exchange and promotes amyloid β plaque formation in mice. Alzheimers Res Ther. 2022;14:59. 10.1186/s13195-022-00999-5. 35473943 10.1186/s13195-022-00999-5 PMC9040291

[156] Hussain R, Tithof J, Wang W, Cheetham-West A, Song W, Peng W, et al. Potentiating glymphatic drainage minimizes post-traumatic cerebral oedema. Nature. 2023;623(7989):992–1000. 10.1038/s41586-023-06737-7. 37968397 10.1038/s41586-023-06737-7 PMC11216305

[157] Xu D, Yang L, Jiang B, Yu X. Glymphatic dysfunction in Moyamoya disease: the influence of arterial stenosis and ventricular enlargement. Neuroimage. 2025;310:121143. 10.1016/j.neuroimage.2025.121143. 40089223 10.1016/j.neuroimage.2025.121143

[158] Harrison IF, Ismail O, Machhada A, Colgan N, Ohene Y, Nahavandi P, et al. Impaired glymphatic function and clearance of tau in an Alzheimer’s disease model. Brain. 2020;143(8):2576–93. 10.1093/brain/awaa179. 32705145 10.1093/brain/awaa179 PMC7447521

[159] Chao X, Fang Y, Lu Z, et al. Impairments of neurovascular coupling after stroke lower glymphatic system function and lead to depressive symptom: a longitudinal cohort study. J Affect Disord. 2024;367:255–62. 10.1016/j.jad.2024.08.229. 39236880 10.1016/j.jad.2024.08.229

[160] Preziosa P, Pagani E, Margoni M, Rubin M, Storelli L, Corazzolla G, et al. Glymphatic system may mediate the relation between choroid plexus and brain damage in multiple sclerosis. Neurol Neuroimmunol Neuroinflamm. 2025;12(4):e200414. 10.1212/NXI.0000000000200414. 40472291 10.1212/NXI.0000000000200414 PMC12153948

[161] Liu K, Zhu J, Chang Y, Lin Z, Shi Z, Li X, et al. Attenuation of cerebral edema facilitates recovery of glymphatic system function after status epilepticus. JCI Insight. 2021;6(17):e151835. 10.1172/jci.insight.151835. 34494549 10.1172/jci.insight.151835 PMC8492308

[162] Seger A, Ophey A, Heitzmann W, Doppler CEJ, Lindner MS, Brune C, et al. Evaluation of a structured screening assessment to detect isolated rapid eye movement sleep behavior disorder. Mov Disord. 2023;38(6):990–9. 10.1002/mds.29389. 37071758 10.1002/mds.29389

[163] Song YM, Jeong J, de Los Reyes AA 5th, Lim D, Cho CH, Yeom JW, et al. Causal dynamics of sleep, circadian rhythm, and mood symptoms in patients with major depression and bipolar disorder: insights from longitudinal wearable device data. EBioMedicine. 2024;103:105094 10.1016/j.ebiom.2024.105094 10.1016/j.ebiom.2024.105094 PMC11002811 38579366

[164] Sangalli L, Boggero IA. The impact of sleep components, quality and patterns on glymphatic system functioning in healthy adults: a systematic review. Sleep Med Rev. 2023;101:322–49. 10.1016/j.sleep.2022.11.012. 10.1016/j.sleep.2022.11.012 36481512

[165] Deng S, Hu Y, Chen S, Xue Y, Yao D, Sun Q, et al. Chronic sleep fragmentation impairs brain interstitial clearance in young wildtype mice. J Cereb Blood Flow Metab. 2024;44(9):1515–31. 10.1177/0271678X241230188. 38639025 10.1177/0271678X241230188 PMC11418708

[166] Du X, Wang H, Liu S, Song Y, Chen X, Chen Z, et al. Astrocytic GluN2A alleviates sleep deprivation-induced elevation of Aβ through regulating neprilysin and AQP4 via the calcineurin/NFAT pathway. Prog Neurobiol. 2025;248:102744. 10.1016/j.pneurobio.2025.102744. 40032156 10.1016/j.pneurobio.2025.102744

[167] Di H, Guo Y, Daghlas I, Wang L, Liu G, Pan A, et al. Evaluation of sleep habits and disturbances among US adults, 2017–2020. JAMA Netw Open. 2022;5(11):e2240788. 10.1001/jamanetworkopen.2022.40788. 36346632 10.1001/jamanetworkopen.2022.40788 PMC9644264

[168] Gardiner C, Weakley J, Burke LM, Roach GD, Sargent C, Maniar N, et al. The effect of alcohol on subsequent sleep in healthy adults: a systematic review and meta-analysis. Sleep Med Rev. 2025;80:102030. 10.1016/j.smrv.2024.102030. 39631226 10.1016/j.smrv.2024.102030

[169] Wu X, Miller JA, Lee BTK, Wang Y, Ruedl C. Reducing microglial lipid load enhances β amyloid phagocytosis in an Alzheimer's disease mouse model. Sci Adv. 2025;11(6):eadq6038. 10.1126/sciadv.adq6038. 10.1126/sciadv.adq6038 PMC11797491 39908361

[170] Fang R, Jin L, Lu H, Xie Q, Yang X, Kueckelhaus M. A novel microsurgical model of cervical lymph node-to-vein anastomosis (LNVA) for studying brain lymphatic outflow. J Craniofac Surg. 2025;36(6):2160–3. 10.1097/SCS.0000000000011535. 40531514 10.1097/SCS.0000000000011535

[171] Wu KM, Xu QH, Liu YQ, Feng YW, Han SD, Zhang YR, et al. Neuronal FAM171A2 mediates α-synuclein fibril uptake and drives Parkinson’s disease. Science. 2025;387(6736):892–900. 10.1126/science.adp3645. 39977508 10.1126/science.adp3645

[172] Ma XR, Prudencio M, Koike Y, Vatsavayai SC, Kim G, Harbinski F, et al. TDP-43 represses cryptic exon inclusion in the FTD-ALS gene UNC13A. Nature. 2022Mar;603(7899):124–30. 10.1038/s41586-022-04424-7. 10.1038/s41586-022-04424-7 PMC8891019 35197626

[173] Bunting EL, Donaldson J, Cumming SA, Olive J, Broom E, Miclăuș M, et al. Antisense oligonucleotide-mediated MSH3 suppression reduces somatic CAG repeat expansion in Huntington's disease iPSC-derived striatal neurons. Sci Transl Med. 2025;17(785):eadn4600. 10.1126/scitranslmed.adn4600. 10.1126/scitranslmed.adn4600 39937881

[174] Liu H, Chen L, Zhang C, Liu C, Li Y, Cheng L, et al. Glymphatic influx and clearance are perturbed in Huntington’s disease. JCI Insight. 2024;9(20):e172286. 10.1172/jci.insight.172286. 39226105 10.1172/jci.insight.172286 PMC11530125

[175] Scialò C, Zhong W, Jagannath S, Wilkins O, Caredio D, Hruska-Plochan M, et al. Seeded aggregation of TDP-43 induces its loss of function and reveals early pathological signatures. Neuron. 2025;113(10):1614-28.e11. 10.1016/j.neuron.2025.03.008. 40157355 10.1016/j.neuron.2025.03.008

[176] Yu Y, Liu X, Zang Z, Zhao X, Zhao B, Zhang Y, et al. Enhanced meningeal lymphatic drainage alleviates cognitive dysfunction induced by anesthesia and surgery in aged mice. Neuropharmacology. 2025;280:110674. 10.1016/j.neuropharm.2025.110674. 40902879 10.1016/j.neuropharm.2025.110674

[177] Tian X, Sinclair DA. Restricting mealtime ameliorates neurodegeneration. Cell Metab. 2023;35(10):1673–4. 10.1016/j.cmet.2023.09.006. 37793341 10.1016/j.cmet.2023.09.006

[178] Whittaker DS, Akhmetova L, Carlin D, Romero H, Welsh DK, Colwell CS, et al. Circadian modulation by time-restricted feeding rescues brain pathology and improves memory in mouse models of Alzheimer’s disease. Cell Metab. 2023;35(10):1704-1721.e6. 10.1016/j.cmet.2023.07.014. 37607543 10.1016/j.cmet.2023.07.014 PMC10591997

[179] Ciampi C, Fagiani F, Murtaj V, Comella F, Torre V, Filibian M, et al. Intestinal inflammation induces glymphatic remodeling, priming early neurodegenerative signals in male mice. Alzheimers Dement. 2025;21(10):e70640. 10.1002/alz.70640. 41065132 10.1002/alz.70640 PMC12509038

[180] Lish AM, Grogan EFL, Benoit CR, Pearse RV 2nd, Heuer SE, Luquez T, et al. CLU alleviates Alzheimer’s disease-relevant processes by modulating astrocyte reactivity and microglia-dependent synaptic density. Neuron. 2025;113(12):1925-1946.e11. 10.1016/j.neuron.2025.03.034. 40311610 10.1016/j.neuron.2025.03.034 PMC12181066

[181] Gulen MF, Samson N, Keller A, Schwabenland M, Liu C, Glück S, et al. cGAS-STING drives ageing-related inflammation and neurodegeneration. Nature. 2023Aug;620(7973):374–80. 10.1038/s41586-023-06373-1. 10.1038/s41586-023-06373-1 PMC10412454 37532932

[182] Reid MM, Menon S, Liu H, Zhou H, Hu Z, Frerich S, et al. Human brain vascular multi-omics elucidates disease-risk associations. Neuron. 2025;113(19):3143-3161.e5. 10.1016/j.neuron.2025.07.001. 40730185 10.1016/j.neuron.2025.07.001 PMC12321221

[183] Gaberel T, Gakuba C, Goulay R, Martinez De Lizarrondo S, Hanouz JL, Emery E, et al. Impaired glymphatic perfusion after strokes revealed by contrast-enhanced MRI: a new target for fibrinolysis? Stroke. 2014;45(10):3092–6. 10.1161/STROKEAHA.114.006617. 10.1161/STROKEAHA.114.006617 25190438

[184] Zhu J, Mo J, Liu K, Chen Q, Li Z, He Y, et al. Glymphatic system impairment contributes to the formation of brain edema after ischemic stroke. Stroke. 2024;55(5):1393–404. 10.1161/STROKEAHA.123.045941. 38533660 10.1161/STROKEAHA.123.045941

[185] Dozono K, Ishii N, Nishihara Y, Horie A. An autopsy study of the incidence of lacunes in relation to age, hypertension, and arteriosclerosis. Stroke. 1991Aug;22(8):993–6. 10.1161/01.str.22.8.993. 10.1161/01.str.22.8.993 1866767

[186] Cao Y, Huang MY, Mao CH, Wang X, Xu YY, Qian XJ, et al. Arteriolosclerosis differs from venular collagenosis in relation to cerebrovascular parenchymal damages: an autopsy-based study. Stroke Vasc Neurol. 2023;8(4):267–75. 10.1136/svn-2022-001924. 36581493 10.1136/svn-2022-001924 PMC10512076

[187] Hong H, Tozer DJ, Chen Y, Brown RB, Low A, Markus HS. Perivascular space dysfunction in cerebral small vessel disease is related to neuroinflammation. Brain. 2025;148(5):1540–50. 10.1093/brain/awae357. 39509331 10.1093/brain/awae357 PMC12073995

[188] Perosa V, Oltmer J, Munting LP, Freeze WM, Auger CA, Scherlek AA, et al. Perivascular space dilation is associated with vascular amyloid-β accumulation in the overlying cortex. Acta Neuropathol. 2022;143(3):331–48. 10.1007/s00401-021-02393-1. 34928427 10.1007/s00401-021-02393-1 PMC9047512

[189] Zhang W, Zhou Y, Wang J, Gong X, Chen Z, Zhang X, et al. Glymphatic clearance function in patients with cerebral small vessel disease. Neuroimage. 2021;238:118257. 10.1016/j.neuroimage.2021.118257. 34118396 10.1016/j.neuroimage.2021.118257

[190] Hassett L. Physiotherapy management of moderate-to-severe traumatic brain injury. J Physiother. 2023;69(3):141–7. 10.1016/j.jphys.2023.05.015. 37286387 10.1016/j.jphys.2023.05.015

[191] Kowalski RG, Hammond FM, Weintraub AH, Nakase-Richardson R, Zafonte RD, Whyte J, et al. Recovery of consciousness and functional outcome in moderate and severe traumatic brain injury. JAMA Neurol. 2021;78(5):548–57. 10.1001/jamaneurol.2021.0084. 33646273 10.1001/jamaneurol.2021.0084 PMC7922241

[192] Iliff JJ, Chen MJ, Plog BA, Zeppenfeld DM, Soltero M, Yang L, et al. Impairment of glymphatic pathway function promotes tau pathology after traumatic brain injury. J Neurosci. 2014;34(49):16180–93. 10.1523/JNEUROSCI.3020-14.2014. 25471560 10.1523/JNEUROSCI.3020-14.2014 PMC4252540

[193] Braun M, Sevao M, Keil SA, Gino E, Wang MX, Lee J, et al. Macroscopic changes in aquaporin-4 underlie blast traumatic brain injury-related impairment in glymphatic function. Brain. 2024;147(6):2214–29. 10.1093/brain/awae065. 38802114 10.1093/brain/awae065 PMC11146423

[194] Zhang X, Sun B, Li W, Liu T, Li W, Chen B, et al. Enhancing glymphatic transport through angiotensin II type 2 receptor activation promotes neurological recovery after traumatic brain injury. Theranostics. 2025;15(18):9775–92. 10.7150/thno.117743. 41041052 10.7150/thno.117743 PMC12486419

[195] Lv C, Han S, Sha Z, Liu M, Dong S, Zhang C, et al. Cerebral glucagon-like peptide-1 receptor activation alleviates traumatic brain injury by glymphatic system regulation in mice. CNS Neurosci Ther. 2023;29(12):3876–88. 10.1111/cns.14308. 37353947 10.1111/cns.14308 PMC10651945

[196] Pignata A, Frieser D, Gonzalez-Fierro C, Hsiao CC, Engelenburg HJ, Alis M, et al. Tissue-resident memory CD4+ T cells infiltrate the CNS in progressive multiple sclerosis and contribute to chronic autoimmunity in mice. Sci Transl Med. 2025;17(808):eadp8109. 10.1126/scitranslmed.adp8109. 10.1126/scitranslmed.adp8109 40700520

[197] Marjo N, Jussi L, Markus M, Johan R, Saara W, Marcus S, et al. Longitudinal accumulation of glial activation measured by TSPO-PET predicts later brain atrophy in multiple sclerosis. J Neuroinflammation. 2025;22(1):200. 10.1186/s12974-025-03519-y. 40775641 10.1186/s12974-025-03519-y PMC12333212

[198] Rocca MA, Ratzinger S, Preziosa P, Meani A, Gueye M, Vezzulli P, et al. Clinical integration of brain and cord MRI features improves differential diagnosis of multiple sclerosis. J Neurol. 2025;272(6):388. 10.1007/s00415-025-13124-x. 40349257 10.1007/s00415-025-13124-x

[199] Järvelä M, Kananen J, Korhonen V, Huotari N, Ansakorpi H, Kiviniemi V. Increased very low frequency pulsations and decreased cardiorespiratory pulsations suggest altered brain clearance in narcolepsy. Commun Med. 2022.Sept.30;2:122. 10.1038/s43856-022-00187-4. 10.1038/s43856-022-00187-4 PMC9525269 36193214

[200] Song YM, Jeong J, de Los Reyes AA 5th, Lim D, Cho CH, Yeom JW, et al. Causal dynamics of sleep, circadian rhythm, and mood symptoms in patients with major depression and bipolar disorder: insights from longitudinal wearable device data. EBioMedicine. 2024;103:105094. 10.1016/j.ebiom.2024.105094. 10.1016/j.ebiom.2024.105094 PMC11002811 38579366

[201] Sangalli L, Boggero IA. The impact of sleep components, quality and patterns on glymphatic system functioning in healthy adults: a systematic review. Sleep Med. 2023;101:322–49. 10.1016/j.sleep.2022.11.012. 36481512 10.1016/j.sleep.2022.11.012

[202] Wu KM, Xu QH, Liu YQ, Feng YW, Han SD, Zhang YR, et al. Neuronal FAM171A2 mediates α-synuclein fibril uptake and drives Parkinson’s disease. Science. 2025;387(6736):892–900. 10.1126/science.adp3645. 39977508 10.1126/science.adp3645

[203] Ma XR, Prudencio M, Koike Y, Vatsavayai SC, Kim G, Harbinski F, et al. TDP-43 represses cryptic exon inclusion in the FTD-ALS gene UNC13A. Nature. 2022;603(7899):124–30. 10.1038/s41586-022-04424-7. 35197626 10.1038/s41586-022-04424-7 PMC8891019

[204] Bunting EL, Donaldson J, Cumming SA, Olive J, Broom E, Miclăuș M, et al. Antisense oligonucleotide-mediated MSH3 suppression reduces somatic CAG repeat expansion in Huntington’s disease iPSC-derived striatal neurons. Sci Transl Med. 2025;17(785):eadn4600. 10.1126/scitranslmed.adn4600. 39937881 10.1126/scitranslmed.adn4600

[205] Liu H, Chen L, Zhang C, Liu C, Li Y, Cheng L, et al. Glymphatic influx and clearance are perturbed in Huntington’s disease. JCI Insight. 2024;9(20):e172286. 10.1172/jci.insight.172286. 39226105 10.1172/jci.insight.172286 PMC11530125

[206] Whittaker DS, Akhmetova L, Carlin D, Romero H, Welsh DK, Colwell CS, et al. Circadian modulation by time-restricted feeding rescues brain pathology and improves memory in mouse models of Alzheimer’s disease. Cell Metab. 2023;35(10):1704-1721.e6. 10.1016/j.cmet.2023.07.014. 37607543 10.1016/j.cmet.2023.07.014 PMC10591997

[207] Lish AM, Grogan EFL, Benoit CR, Pearse RV 2nd, Heuer SE, Luquez T, et al. CLU alleviates Alzheimer’s disease-relevant processes by modulating astrocyte reactivity and microglia-dependent synaptic density. Neuron. 2025;113(12):1925-1946.e11. 10.1016/j.neuron.2025.03.034. 40311610 10.1016/j.neuron.2025.03.034 PMC12181066

[208] Gulen MF, Samson N, Keller A, Schwabenland M, Liu C, Glück S, et al. cGAS-STING drives ageing-related inflammation and neurodegeneration. Nature. 2023;620(7973):374–80. 10.1038/s41586-023-06373-1. 37532932 10.1038/s41586-023-06373-1 PMC10412454

[209] Gaberel T, Gakuba C, Goulay R, Martinez De Lizarrondo S, Hanouz JL, Emery E, et al. Impaired glymphatic perfusion after strokes revealed by contrast-enhanced MRI: a new target for fibrinolysis? Stroke. 2014;45(10):3092–6. 10.1161/STROKEAHA.114.006617. 25190438 10.1161/STROKEAHA.114.006617

[210] Dozono K, Ishii N, Nishihara Y, Horie A. An autopsy study of the incidence of lacunes in relation to age, hypertension, and arteriosclerosis. Stroke. 1991;22(8):993–6. 10.1161/01.str.22.8.993. 1866767 10.1161/01.str.22.8.993

[211] Pignata A, Frieser D, Gonzalez-Fierro C, Hsiao CC, Engelenburg HJ, Alis M, et al. Tissue-resident memory CD4+ T cells infiltrate the CNS in progressive multiple sclerosis and contribute to chronic autoimmunity in mice. Sci Transl Med. 2025;17(808):eadp8109. 10.1126/scitranslmed.adp8109. 40700520 10.1126/scitranslmed.adp8109

[212] Ye C, Wang S, Niu L, Yang F, Wang G, Wang S, et al. Unlocking potential of oxytocin: improving intracranial lymphatic drainage for Alzheimer’s disease treatment. Theranostics. 2024;14(11):4331–51. 10.7150/thno.98587. 39113801 10.7150/thno.98587 PMC11303076

[213] Wu CH, Kuo Y, Chang FC, Lirng JF, Ling YH, Wang YF, et al. Noninvasive investigations of human glymphatic dynamics in a diseased model. Eur Radiol. 2023;33(12):9087–98. 10.1007/s00330-023-09894-5. 37402004 10.1007/s00330-023-09894-5

[214] Zhu DD, Huang YL, Guo SY, Li N, Yang XW, Sui AR, et al. AQP4 aggravates cognitive impairment in sepsis-associated encephalopathy through inhibiting Nav 1.6-mediated astrocyte autophagy. Adv Sci. 2023;10(14):e2205862. 10.1002/advs.202205862. 10.1002/advs.202205862 PMC10190498 36922751

[215] Siret C, van Lessen M, Bavais J, Jeong HW, Reddy Samawar SK, Kapupara K, et al. Deciphering the heterogeneity of the Lyve1+ perivascular macrophages in the mouse brain. Nat Commun. 2022;13(1):7366. 10.1038/s41467-022-35166-9. 36450771 10.1038/s41467-022-35166-9 PMC9712536

[216] Pillay P, Manger PR. Order-specific quantitative patterns of cortical gyrification. Eur J Neurosci. 2007;25(9):2705–12. 10.1111/j.1460-9568.2007.05524.x. 17459107 10.1111/j.1460-9568.2007.05524.x

[217] Eidsvaag VA, Enger R, Hansson HA, Eide PK, Nagelhus EA. Human and mouse cortical astrocytes differ in aquaporin-4 polarization toward microvessels. Glia. 2017;65(6):964–73. 10.1002/glia.23138. 28317216 10.1002/glia.23138 PMC5413834

[218] Ren W, Chen J, Wang W, Li Q, Yin X, Zhuang G, et al. Sympathetic nerve-enteroendocrine L cell communication modulates GLP-1 release, brain glucose utilization, and cognitive function. Neuron. 2024;112(6):972-990.e8. 10.1016/j.neuron.2023.12.012. 38242116 10.1016/j.neuron.2023.12.012

[219] Tian S, Zhou S, Wu W, Lin Y, Wang T, Sun H, et al. GLP-1 receptor agonists alleviate diabetic kidney injury via β-Klotho-mediated ferroptosis inhibition. Adv Sci (Weinh). 2025;12(4):e2409781. 10.1002/advs.202409781. 39630101 10.1002/advs.202409781 PMC11775532

[220] Yun SP, Kam TI, Panicker N, Kim S, Oh Y, Park JS, et al. Block of A1 astrocyte conversion by microglia is neuroprotective in models of Parkinson’s disease. Nat Med. 2018;24(7):931–8. 10.1038/s41591-018-0051-5. 29892066 10.1038/s41591-018-0051-5 PMC6039259

[221] Lv M, Xue G, Cheng H, Meng P, Lian X, Hölscher C, et al. The GLP-1/GIP dual-receptor agonist DA5-CH inhibits the NF-κB inflammatory pathway in the MPTP mouse model of Parkinson’s disease more effectively than the GLP-1 single-receptor agonist NLY01. Brain Behav. 2021;11(8):e2231. 10.1002/brb3.2231. 34125470 10.1002/brb3.2231 PMC8413783

[222] Park JS, Kam TI, Lee S, Park H, Oh Y, Kwon SH, et al. Blocking microglial activation of reactive astrocytes is neuroprotective in models of Alzheimer’s disease. Acta Neuropathol Commun. 2021;9(1):78. 10.1186/s40478-021-01180-z. 33902708 10.1186/s40478-021-01180-z PMC8074239

[223] McGarry A, Rosanbalm S, Leinonen M, Olanow CW, To D, Bell A, et al. Safety, tolerability, and efficacy of NLY01 in early untreated Parkinson’s disease: a randomised, double-blind, placebo-controlled trial. Lancet Neurol. 2024;23(1):37–45. 10.1016/S1474-4422(23)00378-2. 38101901 10.1016/S1474-4422(23)00378-2

[224] Drews HJ, Yenkoyan K, Lourhmati A, Buadze M, Kabisch D, Verleysdonk S, et al. Intranasal losartan decreases perivascular beta amyloid, inflammation, and the decline of neurogenesis in hypertensive rats. Neurotherapeutics. 2019;16(3):725–40. 10.1007/s13311-019-00723-6. 30796737 10.1007/s13311-019-00723-6 PMC6694377

[225] Liu X, Hao J, Yao E, Cao J, Zheng X, Yao D, et al. Polyunsaturated fatty acid supplement alleviates depression-incident cognitive dysfunction by protecting the cerebrovascular and glymphatic systems. Brain Behav Immun. 2020;89:357–70. 10.1016/j.bbi.2020.07.022. 32717402 10.1016/j.bbi.2020.07.022

[226] Zhang R, Wang L, Zhang L, Chen J, Zhu Z, Zhang Z, et al. Nitric oxide enhances angiogenesis via the synthesis of vascular endothelial growth factor and cGMP after stroke in the rat. Circ Res. 2003;92(3):308–13. 10.1161/01.res.0000056757.93432.8c. 12595343 10.1161/01.res.0000056757.93432.8c

[227] Stacey BS, Marley CJ, Tsukamoto H, Dawkins TG, Owens TS, Calverley TA, et al. Phosphodiesterase inhibition restores hypoxia-induced cerebrovascular dysfunction subsequent to improved systemic redox homeostasis: a randomized, double-blind, placebo-controlled crossover study. J Cereb Blood Flow Metab. 2025;45(7):1343–56. 10.1177/0271678X251313747. 39862172 10.1177/0271678X251313747 PMC11765346

[228] Webb AJS, Birks JS, Feakins KA, Lawson A, Dawson J, Rothman AMK, et al. Cerebrovascular effects of sildenafil in small vessel disease: the OxHARP trial. Circ Res. 2024;135(2):320–31. 10.1161/CIRCRESAHA.124.324327. 38832504 10.1161/CIRCRESAHA.124.324327 PMC11227301

[229] Riis TS, Feldman DA, Losser AJ, Okifuji A, Kubanek J. Noninvasive targeted modulation of pain circuits with focused ultrasonic waves. Pain. 2024;165(12):2829–39. 10.1097/j.pain.0000000000003322. 39073370 10.1097/j.pain.0000000000003322 PMC11562753

[230] Xiao H, Sreejith A, Howson I, Cruz AA, Key T, Iliff JJ, et al. Focused ultrasound enhances glymphatic transport robustly across anesthesia levels. Ultrasound Med Biol. 2025;51(10):1701–9. 10.1016/j.ultrasmedbio.2025.06.009. 40653395 10.1016/j.ultrasmedbio.2025.06.009

[231] Gladwell RA, Fisher DG, Wythe JD, Highley CB, Lukens JR, Price RJ. Computational fluid dynamic model prediction of enhanced glymphatic clearance in response to focused ultrasound-mediated blood-brain barrier opening. Adv Sci. 2025. 10.1002/advs.202510684. 10.1002/advs.202510684 PMC12667546 40908557

[232] Gong Y, Xu K, Ye D, Yang Y, Miller MJ, Feng Z, et al. In vivo two-photon microscopy imaging of focused ultrasound-mediated glymphatic transport in the mouse brain. J Cereb Blood Flow Metab. 2025;45(7):1281–92. 10.1177/0271678X251323369. 39985197 10.1177/0271678X251323369 PMC11846094

[233] Hsiao MY, Lin YL, Lin MT, Liao WH, Chen WS, Wu CH. Restoration of glymphatic influx after photothrombotic stroke using low-intensity focused ultrasound. CNS Neurosci Ther. 2025;31(6):e70451. 10.1111/cns.70451. 40457518 10.1111/cns.70451 PMC12129706

[234] Lee Y, Choi Y, Park EJ, Kwon S, Kim H, Lee JY, et al. Improvement of glymphatic-lymphatic drainage of beta-amyloid by focused ultrasound in Alzheimer’s disease model. Sci Rep. 2020;10(1):16144. 10.1038/s41598-020-73151-8. 32999351 10.1038/s41598-020-73151-8 PMC7527457

[235] Mehta RI, Carpenter JS, Mehta RI, Haut MW, Wang P, Ranjan M, et al. Ultrasound-mediated blood-brain barrier opening uncovers an intracerebral perivenous fluid network in persons with Alzheimer’s disease. Fluids Barriers CNS. 2023;20(1):46. 10.1186/s12987-023-00447-y. 37328855 10.1186/s12987-023-00447-y PMC10276371

[236] Wu W, Zhao Y, Cheng X, Xie X, Zeng Y, Tao Q, et al. Modulation of glymphatic system by visual circuit activation alleviates memory impairment and apathy in a mouse model of Alzheimer’s disease. Nat Commun. 2025;16(1):63. 10.1038/s41467-024-55678-w. 39747869 10.1038/s41467-024-55678-w PMC11696061

[237] Han S, Li XX, Wei S, Zhao D, Ding J, Xu Y, et al. Orbitofrontal cortex-hippocampus potentiation mediates relief for depression: a randomized double-blind trial and TMS-EEG study. Cell Rep Med. 2023;4(6):101060. 10.1016/j.xcrm.2023.101060. 37263267 10.1016/j.xcrm.2023.101060 PMC10313932

[238] Yang X, Ma L, Fan C, Wang H, Zhang M, Du H, et al. Efficacy and acceptability of brain stimulation for anxiety disorders, OCD, and PTSD: a systematic review and network meta-analysis of randomized controlled trials. J Affect Disord. 2025;370:62–75. 10.1016/j.jad.2024.10.071. 39477076 10.1016/j.jad.2024.10.071

[239] Lin Y, Jin J, Lv R, Luo Y, Dai W, Li W, et al. Repetitive transcranial magnetic stimulation increases the brain’s drainage efficiency in a mouse model of Alzheimer’s disease. Acta Neuropathol Commun. 2021;9(1):102. 10.1186/s40478-021-01198-3. 34078467 10.1186/s40478-021-01198-3 PMC8170932

[240] Gao X, Wang Y, Chen X, Gao S. Interface, interaction, and intelligence in generalized brain-computer interfaces. Trends Cogn Sci. 2021;25(8):671–84. 10.1016/j.tics.2021.04.003. 34116918 10.1016/j.tics.2021.04.003

[241] Tang J, LeBel A, Jain S, Huth AG. Semantic reconstruction of continuous language from non-invasive brain recordings. Nat Neurosci. 2023;26(5):858–66. 10.1038/s41593-023-01304-9. 37127759 10.1038/s41593-023-01304-9 PMC11304553

[242] Khalil K, Asgher U, Ayaz Y. Novel fNIRS study on homogeneous symmetric feature-based transfer learning for brain–computer interface. Sci Rep. 2022;12:3198. 10.1038/s41598-022-06805-4. 35210460 10.1038/s41598-022-06805-4 PMC8873341

[243] Hauser SL, Zielman R, Das Gupta A, Xi J, Stoneman D, Karlsson G, et al. Efficacy and safety of four-year ofatumumab treatment in relapsing multiple sclerosis: the ALITHIOS open-label extension. Mult Scler. 2023;29(11–12):1452–64. 10.1177/13524585231195346. 37691530 10.1177/13524585231195346 PMC10580679

[244] Birbeck GL, Seydel KB, Mwanza S, Tembo D, Chilombe M, Watts A, et al. Acetaminophen and ibuprofen in pediatric central nervous system malaria: a randomized clinical trial. JAMA Neurol. 2024;81(8):857–65. 10.1001/jamaneurol.2024.1677. 38857015 10.1001/jamaneurol.2024.1677 PMC11165415

[245] Lin PY, Cheng C, Satyanarayanan SK, Chiu LT, Chien YC, Chuu CP, et al. Omega-3 fatty acids and blood-based biomarkers in Alzheimer’s disease and mild cognitive impairment: a randomized placebo-controlled trial. Brain Behav Immun. 2022;99:289–98. 10.1016/j.bbi.2021.10.014. 34755655 10.1016/j.bbi.2021.10.014

[246] Clarke N, Thornton P, Reader V, Lindsay N, Digby Z, Mullen B, et al. Anti-neuroinflammatory and anti-inflammatory effects of the NLRP3 inhibitor NT-0796 in subjects with Parkinson’s disease. Mov Disord. 2025;40(10):2199–208. 10.1002/mds.30307. 40792655 10.1002/mds.30307 PMC12553988

[247] Wang J, Huffman D, Ajwad A, McLouth CJ, Bachstetter A, Kohler K, et al. Thermoneutral temperature exposure enhances slow-wave sleep with a correlated improvement in amyloid pathology in a triple-transgenic mouse model of Alzheimer’s disease. Sleep. 2024;47(6):zsae078. 10.1093/sleep/zsae078. 38512801 10.1093/sleep/zsae078 PMC13032130

[248] Benjafield AV, Sert Kuniyoshi FH, Malhotra A, Martin JL, Morin CM, Maurer LF, et al. Estimation of the global prevalence and burden of insomnia: a systematic literature review-based analysis. Sleep Med Rev. 2025;82:102121. 10.1016/j.smrv.2025.102121. 40627924 10.1016/j.smrv.2025.102121 PMC12676268

[249] Yeung WF, Lai AY, Yu BY, Ho FY, Chung KF, Ho JY, et al. Effect of zero-time exercise on physically inactive adults with insomnia disorder: a randomized controlled trial. Int J Nurs Stud. 2025;165:105033. 10.1016/j.ijnurstu.2025.105033. 39999728 10.1016/j.ijnurstu.2025.105033

[250] Wallace DA, Reid K, Grobman WA, Facco FL, Silver RM, Pien GW, et al. Associations between evening shift work, irregular sleep timing, and gestational diabetes in the Nulliparous Pregnancy Outcomes Study: Monitoring Mothers-to-be (nuMoM2b). Sleep. 2023;46(4):zsac297. 10.1093/sleep/zsac297. 36477807 10.1093/sleep/zsac297 PMC10091083

[251] Merino D, Gérard AO, Van Obberghen EK, Ben Othman N, Ettore E, Giordana B, et al. Medications as a trigger of sleep-related eating disorder: a disproportionality analysis. J Clin Med. 2022;11(13):3890. 10.3390/jcm11133890. 35807172 10.3390/jcm11133890 PMC9267629

[252] Chhe K, Hegde MS, Taylor SR, Farkas ME. Circadian effects of melatonin receptor-targeting molecules in vitro. Int J Mol Sci. 2024;25(24):13508. 10.3390/ijms252413508. 39769270 10.3390/ijms252413508 PMC11727910

[253] Yoo RE, Kim JH, Moon HY, Park JY, Cheon S, Shin HS, et al. Long-term physical exercise facilitates putative glymphatic and meningeal lymphatic vessel flow in humans. Nat Commun. 2025;16(1):3360. 10.1038/s41467-025-58726-1. 40204790 10.1038/s41467-025-58726-1 PMC11982307

[254] Talbot JS, Perkins DR, Dawkins TG, Douglas AJM, Griffiths TD, Richards CT, et al. Neurovascular coupling and cerebrovascular hemodynamics are modified by exercise training status at different stages of maturation during youth. Am J Physiol Heart Circ Physiol. 2023;325(3):H510–21. 10.1152/ajpheart.00302.2023. 37450291 10.1152/ajpheart.00302.2023 PMC10538977

[255] Mandal G, Alboni S, Cattane N, Marizzoni M, Saleri S, Arslanovski N, et al. The dietary ligands, omega-3 fatty acid endocannabinoids and short-chain fatty acids prevent cytokine-induced reduction of human hippocampal neurogenesis and alter the expression of genes involved in neuroinflammation and neuroplasticity. Mol Psychiatry. 2025;30(11):5338–55. 10.1038/s41380-025-03119-5. 40670679 10.1038/s41380-025-03119-5 PMC12532594

[256] Yang C, Tian S, Liu M, Tan F, Wang W, Du W, et al. Association of MRI parameters of brain waste clearance function with cortisol and inflammatory markers in middle-aged and older adults with major depressive disorder. Asian J Psychiatr. 2025;112:104667. 10.1016/j.ajp.2025.104667. 40885121 10.1016/j.ajp.2025.104667

[257] Peng W, Yuan Y, Lei J, Zhao Y, Li Y, Qu Q, et al. Long-term high-fat diet impairs AQP4-mediated glymphatic clearance of amyloid beta. Mol Neurobiol. 2025;62(1):1079–93. 10.1007/s12035-024-04320-3. 38958889 10.1007/s12035-024-04320-3

[258] Schirge PM, Perneczky R, Taoka T, Ruiz-Rizzo AL, Ersoezlue E, Forbrig R, et al. Perivascular space and white matter hyperintensities in Alzheimer’s disease: associations with disease progression and cognitive function. Alzheimers Res Ther. 2025;17(1):62. 10.1186/s13195-025-01707-9. 40098158 10.1186/s13195-025-01707-9 PMC11917016

[259] Cai X, Sun W, Cai M, Li D, Chen Z, Li H, et al. Impaired glymphatic function contributes to high-frequency attacks in patients with episodic migraine. J Headache Pain. 2025;26(1):132. 10.1186/s10194-025-02070-8. 40461958 10.1186/s10194-025-02070-8 PMC12135552

[260] Chen CL, Son SJ, Schweitzer N, Jin H, Li J, Wang L, et al. Periventricular diffusivity reflects APOE ε4-modulated amyloid accumulation and cognitive impairment in the Alzheimer’s disease continuum. Alzheimers Dement. 2025;21(9):e70659. 10.1002/alz.70659. 40965291 10.1002/alz.70659 PMC12444947

[261] Ran L, Fang Y, Cheng C, He Y, Shao Z, Kong Y, et al. Genome-wide and phenome-wide studies provided insights into brain glymphatic system function and its clinical associations. Sci Adv. 2025;11(3):eadr4606. 10.1126/sciadv.adr4606. 39823331 10.1126/sciadv.adr4606 PMC11740961

[262] Ye D, Chen S, Liu Y, Weixel C, Hu Z, Yuan J, et al. Mechanically manipulating glymphatic transport by ultrasound combined with microbubbles. Proc Natl Acad Sci U S A. 2023;120(21):e2212933120. 10.1073/pnas.2212933120. 37186852 10.1073/pnas.2212933120 PMC10214201

[263] Zhang C, Zheng Y, Jiang G, Luo J, Su L, Ai Y, et al. Enhancement of glymphatic function and cognition in chronic insomnia using low-frequency rTMS. Sleep. 2025;48(6):zsaf083. 10.1093/sleep/zsaf083. 40121525 10.1093/sleep/zsaf083

[264] Zhou X, He Y, Xu T, Wu Z, Guo W, Xu X, et al. 40 Hz light flickering promotes sleep through cortical adenosine signaling. Cell Res. 2024;34(3):214–31. 10.1038/s41422-023-00920-1. 38332199 10.1038/s41422-023-00920-1 PMC10907382

[265] Ma Y, Han Y. Targeting the brain’s glymphatic pathway: a novel therapeutic approach for cerebral small vessel disease. Neural Regen Res. 2026;21(2):433–42. 10.4103/NRR.NRR-D-24-00821. 39688573 10.4103/NRR.NRR-D-24-00821 PMC12220683

